# Immunoglobulin divalence promotes B-cell antigen receptor cluster scale-dependent functions

**DOI:** 10.1038/s41423-025-01327-1

**Published:** 2025-08-06

**Authors:** Erdem Yilmaz, Amirmohammad Rahimi, Matthias Münchhalfen, Mihai Alevra, Arash Golmohammadi, Christian Tetzlaff, Felipe Opazo, Niklas Engels

**Affiliations:** 1 Institute of Cellular & Molecular Immunology, University Medical Center Göttingen, Göttingen, Germany; 2 Center for Biostructural Imaging of Neurodegeneration (BIN), University Medical Center Göttingen, Göttingen, Germany; 3 Institute of Neuro- and Sensory Physiology, University Medical Center Göttingen, Göttingen, Germany; 4 Group of Computational Synaptic Physiology, Department of Neuro- and Sensory Physiology, University Medical Center Göttingen, Göttingen, Germany; 5 NanoTag Biotechnologies GmbH, Göttingen, Germany

**Keywords:** B cell antigen receptor, Immunoglobulin, Signal transduction, super resolution microscopy, B cell activation, B-cell receptor, Calcium signalling

## Abstract

Antibodies, also known as immunoglobulins, share an evolutionarily conserved dimeric core structure with two antigen binding sites. However, recognition of foreign molecules can be achieved by monovalent binding domains, as evidenced by the T-cell antigen receptor and various innate immune receptors. Thus, the reason for the strict evolutionary conservation of immunoglobulin divalence remains unclear. In addition to being soluble immune effector molecules, each immunoglobulin is also expressed as a membrane-bound isoform in the context of the B-cell antigen receptor (BCR). Here, we generated monovalent BCRs and found that their signaling and antigen internalization capabilities were strongly impaired. By using advanced superresolution imaging of BCRs following stimulation with antigens of distinct valences, we showed that the receptor cluster scale in the plasma membrane determines the magnitude of intracellular signaling. The incorporation of additional ITAMs into single BCRs did not increase receptor sensitivity but caused cellular desensitization. Our results demonstrate that the BCR-controlled signaling machinery senses the clustering status of the BCR and that subtle changes in cluster sizes are translated into cellular responses. These findings improve our knowledge of adaptive immune receptor function and will aid in the design of synthetic chimeric antigen receptors.

## Introduction

Antibodies are the universal effector molecules of adaptive humoral immunity in vertebrates. Their basic structure is a dimer of dimers consisting of two identical immunoglobulin (Ig) heavy chains (HCs), each of which is covalently associated with an identical light chain (LC), resulting in their typical [HCLC]_2_ architecture [1]. In some genera, such as camelids and sharks, antibodies can form without LCs [2]. Nonetheless, also these so-called HC antibodies are dimers of identical HCs and thus have two antigen binding sites. The dimeric core of Igs provides these molecules with an avidity that is disproportionately greater than the affinity of single binding sites [3]. The avidity of soluble antibodies can be further increased by di- or multimerization, as observed for secretory IgA and for IgM, respectively. In addition to being produced as soluble immune effector molecules, all Ig isotypes furthermore exist as membrane-bound molecules (mIgs) that are associated with an invariable heterodimer of mIg-associated α and β chains (Igα/β, CD79A/B) [4–6]. The resulting multitransmembrane protein complex consisting of mIg and Igα/β in a 1:1 stoichiometry serves as a B-cell antigen receptor (BCR) that transmits cell fate-determining signals into the cell and mediates efficient endocytosis of bound antigens [7]. The invariable Igα/β chains of the BCR each contain one copy of the immunoreceptor tyrosine-based activation motif (ITAM) in their cytoplasmic domains [8]. Moreover, Igα possesses an additional signaling motif known as tyrosine 204 (Y204), which is located C-terminal of the ITAM [9]. Furthermore, the isotypes mIgG and mIgE contain the immunoglobulin tail tyrosine (ITT) motif in the cytoplasmic tail of each HC [10]. All of these tyrosine motifs are phosphorylated by cytoplasmic protein tyrosine kinases (PTKs) upon BCR activation and then serve as docking sites for intracellular SH2 domain-containing signaling proteins. ITAMs fulfill several roles in the context of BCR function. First, when phosphorylated on both tyrosine residues, they serve as binding sites for the tandem SH2 domains of the PTK Syk and thus recruit this key signaling enzyme to the engaged BCR [11]. Second, they serve as allosteric activators for bound Syk and thus induce a feed-forward loop by increasing the enzyme’s catalytic activity [12]. Third, nonphosphorylated ITAM tyrosines are docking sites for the AP2 adaptor complex, which supports clathrin-mediated receptor endocytosis [13]. In addition, ITAMs regulate the survival of B cells via tonic signaling even in the absence of antigenic stimulation [14].

A key signaling pathway triggered by stimulated BCRs and activated Syk is the influx of the second messenger Ca^2+^ into the cytosol [15]. The kinetics, i.e., the intensity and duration of BCR-induced Ca^2+^ mobilization, are closely connected to B-lymphoid cell fate decisions, such as apoptosis versus survival and cell proliferation [16]. Furthermore, a variety of transcriptional regulators shuttle from the cytosol to the nucleus following BCR ligation. These regulators include members of the NFAT and NF-κB families [17] as well as the microphthalmia family member transcription factor EB (TFEB), which was recently identified as a cell fate-determining element in antigen-activated B cells [18].

In addition to its role in controlling intracellular signaling pathways, the BCR also serves as an efficient endocytosis receptor that targets bound antigens to vesicular compartments of antigen processing, thus promoting MHC class II-based antigenic peptide presentation of protein antigens to T helper cells [19, 20]. T-cell help via cell surface coreceptor interactions and cytokines is particularly important for efficient B-cell activation and differentiation in the context of protein antigens, which often contain only a few identical epitopes. Thus, the differentiation fate of a B-cell is determined by the kinetics of BCR-induced signaling, its ability to present antigenic peptides to T cells, and the influence of either the signal-amplifying or the suppressing activities of coreceptors.

While the investigation of BCR-controlled signaling pathways has reached a reasonable consensus in the field, the mechanism by which the BCR controls its intracellular effector pathways is still a matter of intense debate [21]. Specifically, the status change that a BCR undergoes to switch from inactive to active and how its activity is affected by the physical properties of the antigen remain insufficiently understood. The most prevalent models of BCR activation propose either antigen-induced clustering of monomeric BCRs [22], disaggregation of preexisting BCR clusters [23], or conformational changes within the receptor complex to induce BCR tyrosine-motif phosphorylation and subsequent signaling [24]. Evidence has been provided for each of the partially opposing models [25–28], highlighting the need for novel experimental approaches to address this key aspect of adaptive immunity.

Here, we describe the generation of BCRs with monovalent antigen recognition capacity and test their responsiveness to defined antigens with distinct valences. We revealed the minimal valence requirements of antigens and BCRs to stimulate signaling and endocytosis. Using stoichiometric labeling of single BCRs, stimulated emission depletion (STED) microscopy and image analysis of plasma membrane sheets, we found that subtle changes in cluster formation control the biological activities of this receptor. Even monovalent antigen stimulation caused the formation of small BCR clusters, which, however, did not spark signaling. The addition of additional ITAMs into the BCR did not improve its antigen sensitivity but caused cellular desensitization. Thus, the number of ITAM copies in a given immunoreceptor—two in the case of the BCR versus, e.g., 10 in the case of the T-cell antigen receptor—seems to have adapted to fit the respective cellular signaling machinery and mode of receptor activation to ensure the appropriate activities of the receptors in resting and antigen-exposed lymphocytes.

## Results

### Generation of a heterodimeric mIgG-BCR

**Fig. 1:**
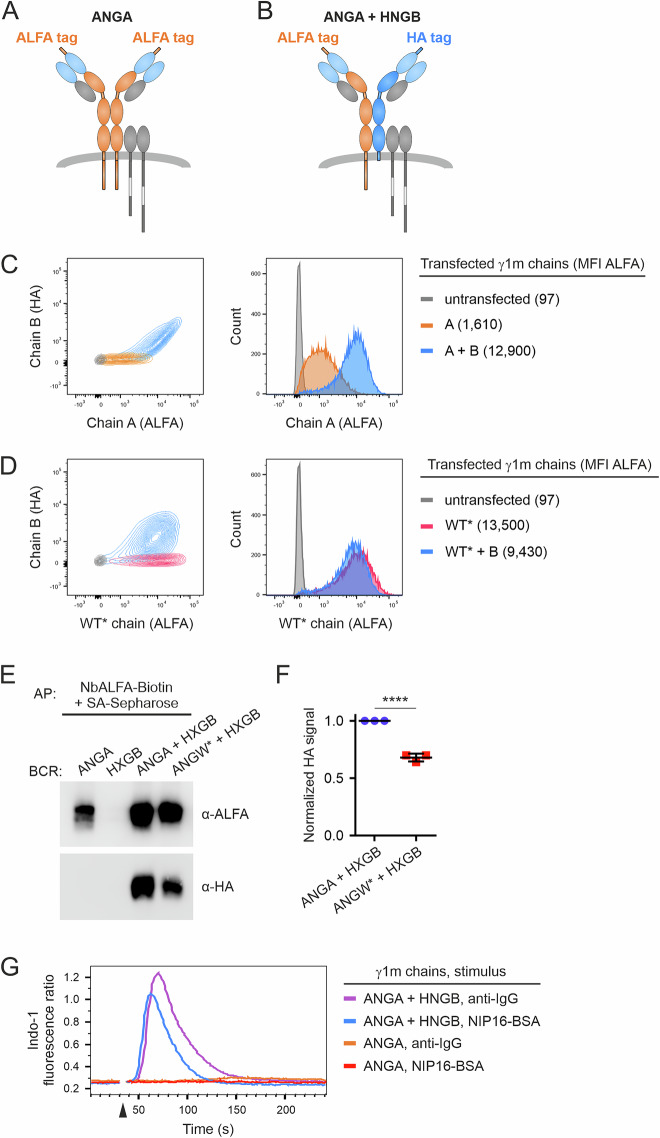
Generation of a heterodimeric mIgG-BCR. **A**, **B** Diagrams of mIgG1-containing BCRs consisting of modified human γ1m immunoglobulin heavy chains. **A** The modified A chain (ANGA, amber-colored) contains an N-terminal ALFA tag and an N(I)P-specific VH domain. **B** NIP-specific BCR consisting of the modified A chain and the modified B chain (in dark blue), which contains an N-terminal HA tag. The NIP-reactive V regions are colored light blue, whereas the Igα/β dimer is shown in gray. **C** Flow cytometric analysis of RHLKO cells expressing the modified A chain alone (amber) or A and B chains together (blue). The cells were stained with reagents binding the ALFA-tag (AF647) and the HA-tag (BV421). **D** RHLKO cells expressing either a γ1m wild-type chain (WT*, see text for details) or the WT* chain and chain B together were analyzed as in (**C**). **E** Affinity purification of mIgG1-BCRs consisting of either one or two different heavy chains. BCRs were purified via the ALFA tag, and the material was probed for the presence of A and B chains by detecting the ALFA and HA tag, respectively. **F** Statistical analysis of band intensity measurements from three independent experiments shown in (**E**). The values for ANGA + HXGB were set to 1.0. The error bars represent the means ± SDs of three independent experiments. Statistical significance was tested in GraphPad Prism software via an unpaired *t* test. ∗∗∗∗*p* ≤ 0.0001. **G** Ca^2+^ mobilization analysis of the BCRs shown in (**A**) and (**B**) after stimulation with either anti-IgG F(ab′)_2_ fragments or NIP16-BSA. The addition of stimulating reagents is indicated by an arrowhead. The data are representative of three independent experiments

The dimeric nature of IgG antibodies has been pharmacologically exploited to generate bispecific therapeutic antibodies, most of which are used in cancer therapy [29]. Several molecular engineering strategies have been developed to obtain heterodimeric IgG molecules with distinct antigen binding sites, such as the knob-in-hole approach or electrostatic steering of the CH3 domain, which contains the γ HC homodimerization interface. Since the IgG CH3 domains in the context of the BCR make contacts with the Igα/β dimer [5], we used the approach described by Von Kreudenstein et al. to generate a human γ1m heterodimeric Fc that retains its natural biophysical properties [30]. In addition, we introduced hinge region mutations [31] to support heterodimerization of mutant γ1m HCs (Supplementary Fig. S1A, B). The two resulting γ1m HCs were termed chain A and chain B, respectively, both of which contained a VH domain specific for the hapten 3-nitro-4-hydroxy-(5-iodo)phenylacetyl (N(I)P). To focus on BCR functions that are exclusively sparked by ITAMs, we inactivated the ITT motif in the A chain and deleted the intracellular tail in the B chain, the latter of which further improved cell surface expression [10]. To distinguish the different chains in biochemical assays, chain A contains an N-terminal ALFA tag [32], whereas chain B contains an HA tag [33] at the same position. We termed the resulting γ1m chains ‘ALFA-NIP-Gamma-A’ (ANGA) and ‘HA-NIP-Gamma-B’ (HNGB), respectively. To express these chains in the context of a BCR, we used a derivative of the human B-cell line Ramos that lacks expression of its endogenous HC and LC (hereafter referred to as RHLKO) [34]. The cells were transduced to express an LC consisting of the VL domain derived from the N(I)P-specific hybridoma B1-8 and the human κ constant domain, resulting in RHLKO/LC cells (Supplementary Fig. S1E, F). These cells were then transduced to express both HC variants either alone or in combination (Fig. 1A, B, Supplementary Figs. S2, S3, see ANGA, HNGB and ANGA + HNGB). Importantly, surface expression of the A chain alone was inefficient, indicating poor homodimerization and/or stability. In contrast, the B chain was readily expressed on the surface of RHLKO/LC cells when it was introduced on its own. Crucuially, in cells that were transduced with both A and B HCs, the A chain appeared on the cell surface almost 10 times more efficiently than in cells expressing the A chain only (Fig. 1C and Supplementary Fig. S2). This shows that the A chain needs a partnering B chain to be expressed as a BCR on the cell surface. This notion is supported by the diagonal shape of the ALFA/HA double-positive cell population, which is a telling sign of heterodimer formation. In control cells that were transduced to express the B chain together with a wild-type chain (in which the ITT motif was inactivated as before and which is thus abbreviated ANGW*, see Supplementary Fig. S1D), the ALFA/HA double-positive population adopted a round shape, indicating that both chains are expressed as homo and heterodimers in a stochastic manner (Fig. 1D and Supplementary Fig. S2). To further test for heterodimerization of the A and B chains, we purified homo and heterodimeric BCRs from Ramos cells using the ALFA tag as a hook and tested for the presence of the B chain by using an anti-HA antibody as detection reagent. The results revealed that cells expressing A and B chains together formed significantly more heterodimers than did cells coexpressing chains W* and B (Fig. 1E, F). Finally, to test whether the heterodimeric BCR was signaling competent, we stimulated cells expressing ANGA + HNGB with either anti-IgG F(ab′)_2_ or polyvalent NIP16-BSA and used intracellular Ca^2+^ mobilization as a readout for BCR activation. Both stimuli caused robust BCR signaling (Fig. 1G). Importantly, cells expressing only the ANGA chain (plus the LC) did not respond to either stimulus, indicating that the small number of cell surface-expressed A chain-only BCRs were nonfunctional (Fig. 1G). In conclusion, the approach to generate heterodimeric mIgG-BCRs on the basis of the introduction of complementary dimerization interfaces in the CH3 domains and accompanying electrostatic mutations in the hinge regions of human γ1m HCs led to successful cell surface expression of functional, antigen-specific BCRs.

### The signaling of monovalent mIgG-BCRs is severely compromised

**Fig. 2:**
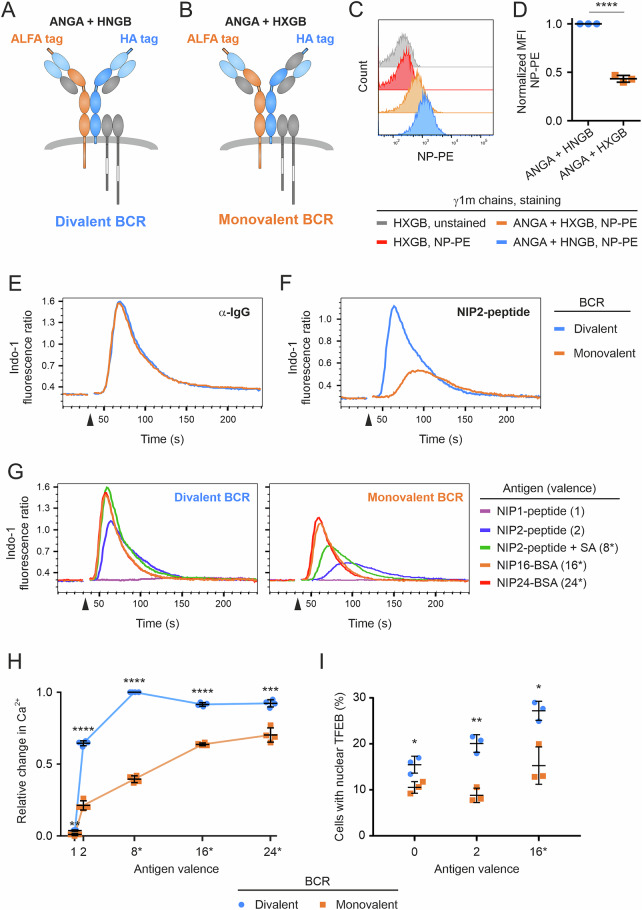
Comparison of divalent and monovalent mIgG-BCRs. **A**, **B** Diagrams of divalent and monovalent mIgG1-BCRs. The divalent BCR in (**A**) contains two N(I)P-specific VH domains, whereas the monovalent BCR in (**B**) contains only one. **C** Flow cytometric analysis of NP binding. RHLKO/LC cells expressing the indicated heavy chains were stained with NP-PE. **D** Statistical analysis of three independent experiments shown in (**C**). The error bars represent the means ± SDs of three independent experiments. Statistical significance was tested in GraphPad Prism software via an unpaired t test. ∗∗∗∗*p *≤ 0.0001. **E** Ca^2+^ mobilization analysis of RHLKO cells expressing either a divalent (blue) or monovalent (amber) BCR upon stimulation with anti-IgG F(ab′)_2_ fragments. **F** The same cells as in (**E**) were stimulated with a peptide containing two NIP groups. **G** The same cells as in (**E**) were stimulated with NIP-containing reagents of various valences. **H** Statistical analysis of the data of four independent experiments shown in (**G**). The maximum change in Indo-1 fluorescence intensity for each stimulation condition is shown. All data points were normalized to the strongest change, which was set to 1.0. The error bars represent the means ± SDs of four independent experiments. Statistical significance was tested via the Holm‒Sidak method, with alpha = 0.05. Each row was analyzed individually without assuming a consistent SD. ∗*p* ≤ 0.05; ∗∗*p* ≤ 0.01; ∗∗∗*p* ≤ 0.001; ∗∗∗∗*p* ≤ 0.0001. **I** Nuclear translocation of TFEB was analyzed by imaging flow cytometry. The cells were left untreated (0) or were stimulated for 60 min at 37 °C with the indicated antigens. The error bars represent the means ± SDs of three independent experiments. Statistical significance was tested in GraphPad Prism software via multiple *t* tests. ∗*p *≤ 0.05; ∗∗*p* ≤ 0.01

Next, we used our system to express heterodimeric mIgG1 to generate conventional divalent and mutant monovalent BCRs. To this end, we inactivated the NIP-reactive VH domain in the B chain, resulting in HXGB (see Supplementary Figs. S1C, S3 and S4A). The expression of this chain together with the ANGA chain resulted in the same diagonally shaped double-positive population as previously observed (Supplementary Fig. S2). Thus, we obtained heterodimeric mIgG-BCRs that are either monovalent or divalent for NIP with similar cell surface expression (Fig. 2A, B and Supplementary Fig. S2). Given the same surface expression of both BCRs, we reasoned that cells expressing the monovalent variant should bind approximately half the amount of antigen as cells expressing the divalent BCRs do. Staining of the cells with NP coupled with fluorescent phycoerythrin (NP-PE) indeed revealed an ~50% reduction in the fluorescence intensity of the cells expressing monovalent mIgG-BCRs (Fig. 2C, D). To compare the signaling capacities of mono- and divalent BCRs, we analyzed their Ca^2+^ mobilization capabilities in response to various stimuli. First, we used anti-IgG F(ab′)_2_ fragments to test the antigen-independent BCR responsiveness of the cells, which was virtually identical in both cell types (Fig. 2E). Next, we used a peptide carrier that contained two NIP groups (NIP2-peptide) to stimulate mono- and divalent BCRs in an antigen-specific manner (titration of the optimal stimulating concentration is shown in Supplementary Fig. S4B). This experiment revealed that monovalent mIgG-BCRs were poorly activated by the divalent antigen, whereas divalent receptors responded faster and with a greater magnitude (Fig. 2F). We extended this analysis to include additional NIP-containing antigens with increasing valences. These included the N-terminally biotinylated NIP2 peptide, which was preincubated with tetrameric streptavidin at a 4:1 molar ratio and thus may have up to eight NIP groups per carrier (since the resulting valence is based on theoretical calculations, it is indicated with 8* in all figures). Furthermore, we used bovine serum albumin conjugated to NIP with a degree of 16 or 24 (NIP16-BSA and NIP24-BSA, respectively) as polyvalent stimuli. Since the coupling ratios of these reagents represent average values of unknown distributions, we indicate the valences with 16* and 24*, respectively. Finally, we included a carrier peptide with only one NIP group (NIP1 peptide). We used all of these reagents to stimulate cells expressing either divalent or monovalent BCRs and assessed their Ca^2+^-mobilizing capacities as previously described (Fig. 2G). The divalent BCR was clearly superior to its monovalent counterpart, as evidenced by faster and more vigorous Ca^2+^ kinetics upon stimulation with every NIP-containing antigen tested. Importantly, the monovalent NIP peptide had no effect on either of the cells. When the Ca^2+^ mobilization capacities of both receptors were plotted against antigen valence, the divalent BCR showed a hyperbolic response characteristic, whereas the monovalent BCR showed an almost linear pattern (Fig. 2H), such that the strongest differences between both receptors were observed upon stimulation with low-valence antigens. However, even the strongest Ca^2+^ response of the monovalent receptor did not reach that of the divalent BCR and remained at the level obtained when divalent BCRs were stimulated with a divalent antigen. We observed very similar response characteristics when we analyzed the phosphorylation of Igα and Syk in the same cells (Supplementary Fig. S4C, D). To test whether these differences in BCR-proximal signaling events impacted more distal effects, we analyzed the nuclear translocation of TFEB by imaging flow cytometry (see Supplementary Fig. S5 for details). Clearly, the monovalent mIgG-BCR was impaired in its ability to induce TFEB translocation, as evidenced by the reduced frequency of cells with nuclear TFEB after 60 min of stimulation with both divalent and polyvalent antigens (Fig. 2I). Collectively, these data show that the evolutionarily conserved divalence of mIgG-BCRs is critical for achieving optimal receptor responsiveness, especially toward low-valence antigens.

### Monovalent mIgM-BCRs are signaling defective

**Fig. 3:**
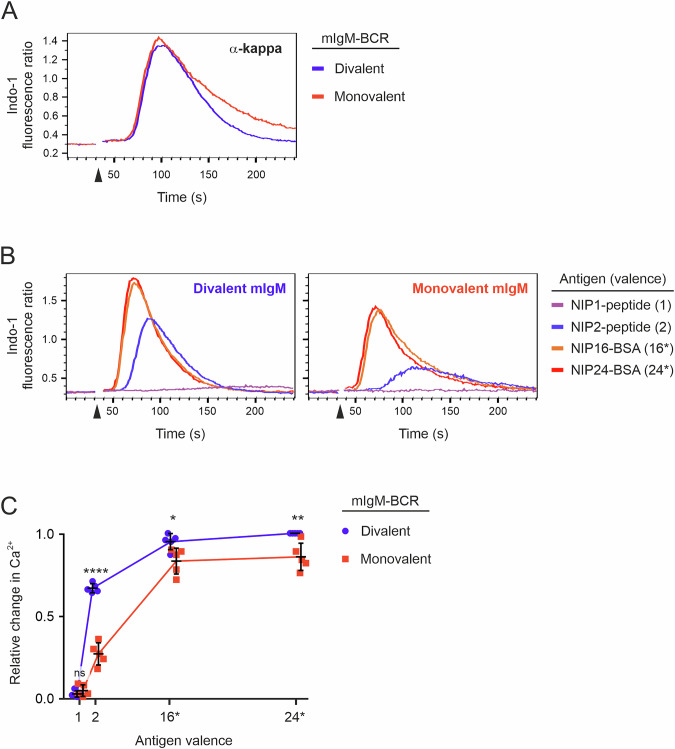
Monovalent mIgM-BCRs are functionally compromised. **A** Ca^2+^ mobilization analysis of RHLKO cells expressing either divalent (blue) or monovalent (red) mIgM-BCR upon stimulation with anti-κ F(ab′)_2_ fragments. **B** The same cells as in (**A**) were stimulated with NIP-containing reagents of various valences. **C** Statistical analysis of three independent experiments shown in (**B**). The error bars represent the means ± SDs of five independent experiments. Statistical significance was tested in GraphPad Prism software via multiple *t* tests. ∗*p* ≤ 0.05; ∗∗*p* ≤ 0.01; ∗∗∗∗*p* ≤ 0.0001; ns not significant

We next investigated whether the observations made with mIgG-BCRs represent a more general functional principle. Thus, we generated monovalent BCRs of the isotype mIgM. The generation of bispecific IgM requires more modifications than does the generation of bispecific IgG since IgM contains two dimerization interfaces, one in the µ CH4 domain and the other in the CH2 domain. We modified both interfaces according to the strategy of Skegro et al. [35] (Supplementary Fig. S6). Following our established nomenclature, we termed the ALFA-tagged chain ANMA, the HA-tagged chain HNMB and the HA-tagged chain having an inactivated NIP-binding site HXMB. When cotransfected into RHLKO/LC cells, both A- and B-µm HCs were expressed on their own on the cell surface (Supplementary Fig. S7A). However, we again observed that the population of double-positive cells had a diagonal shape, demonstrating proportional surface expression of A and B chains, which is typical for heterodimers. Following cell sorting, we obtained pure populations of cells expressing either monovalent or divalent mIgM (Supplementary Fig. S7A). Compared with cells expressing divalent mIgM-BCRs (which have twice the number of binding sites), cells stained with the monovalent NIP1 peptide bound only ~50% of the antigen (Supplementary Fig. S7B). As before, the general BCR signaling capacities of the two cell lines were very similar, as tested by stimulation with F(ab′)_2_ fragments against the LC (Fig. 3A). When stimulated with low- and high-valence antigens, the mono- and divalent mIgM-expressing cells presented similar responses as previously observed in cells expressing mono- or divalent mIgG, with divalent mIgM being superior to its monovalent counterpart (Fig. 3B, C). Thus, the dimeric nature of mIgs, which provides BCRs with two identical antigen binding sites, equips the receptors with greatly increased sensitivity irrespective of the isotype. This was especially true for low-valence antigens, as they are found in small aggregates of protein antigens [36].

### BCR internalization requires polyvalent interactions

**Fig. 4:**
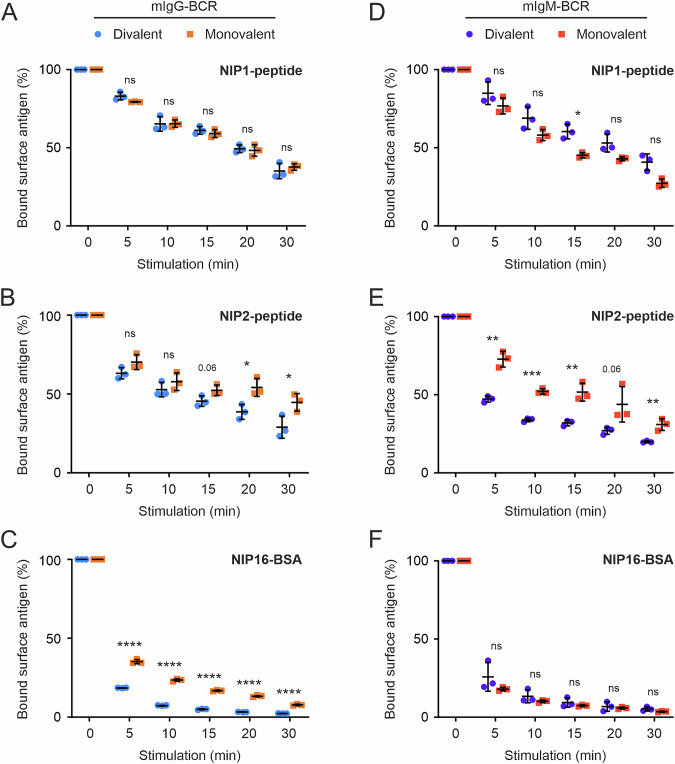
Efficient internalization of antigen-activated BCRs requires immunoglobulin divalence. Kinetics of antigen-induced internalization of divalent and monovalent NIP-reactive mIgG-BCRs (**A**–**C**) and mIgM-BCRs (**D**–**F**). The cells were incubated either with a monovalent NIP1 peptide (**A**, **D**), a divalent NIP2 peptide (**B**, **E**) or with polyvalent NIP16-BSA (**C**, **F**) for the indicated time periods at 37 °C. The remaining cell-surface bound antigen was detected by staining residual cell-surface biotinylated antigens with streptavidin-APC, and the data were plotted as percentages of the surface antigen. Cells incubated on ice during the entirety of the experiment served as controls (time point 0). Streptavidin-APC fluorescence intensities were analyzed via flow cytometry. The mean fluorescence intensities (MFIs) of the control cells were set to 100% for each cell line, and all other values were normalized accordingly. The error bars represent the means ± SDs of three independent experiments. Statistical significance was tested in GraphPad Prism software via multiple t tests. ∗*p* ≤ 0.05; ∗∗*p* ≤ 0.01; ∗∗∗*p *≤ 0.001; ∗∗∗∗*p* ≤ 0.0001; ns not significant

In addition to controlling and initiating intracellular signaling pathways, the BCR serves as an endocytosis receptor for bound antigens. To test the valence requirements for efficient antigen internalization, we used RHLKO cells expressing either di- or monovalent mIgG-BCRs, incubated them with NIP-based antigens of distinct valences (all of which were biotinylated) and measured receptor endocytosis by staining residual surface antigens with fluorescent streptavidin (see Supplementary Fig. S8 for details). The signaling-inert NIP1 peptide served as a control to establish steady-state BCR endocytosis rates. As expected, both mono- and divalent mIgG-BCRs presented the same steady-state endocytosis kinetics, with ~60% of the receptors being internalized within 30 min at 37 °C (Fig. 4A). When the NIP2 peptide was used, the internalization rate of monovalent BCRs was practically indistinguishable from the steady-state rate, whereas that of divalent BCRs was moderately accelerated (Fig. 4B), indicating that moderate BCR signaling does not efficiently promote antigen internalization. When the cells were incubated with NIP16-BSA, the monovalent BCR was endocytosed much faster than observed with low-valence antigens, resulting in ~60% internalization after 5 min of incubation (Fig. 4C). However, the divalent BCR was internalized even more efficiently when stimulated with the polyvalent antigen at every time point tested in our kinetics analyses, resulting in approximately 80% internalization after 5 min at 37 °C (Fig. 4C). We also studied antigen internalization by mono- and divalent mIgM-BCRs upon stimulation with the same reagents, which resulted in similar overall kinetic patterns (Fig. 4D–F). However, the divalence of mIgM-BCRs was particularly important for the internalization of divalent antigens (Fig. 4E), while there was no difference in antigen internalization between mono- and divalent mIgM when incubated with polyvalent NIP16-BSA (Fig. 4F). These findings indicate that mIgG and mIgM-BCRs may have distinct internalization requirements. In summary, the evolutionarily conserved divalent nature of BCRs not only increases their sensitivity to low-valence antigens but also supports efficient antigen internalization, promoting peptide presentation by activated B cells to T cells.

### The magnitude of BCR signaling is determined by the BCR cluster size

**Fig. 5:**
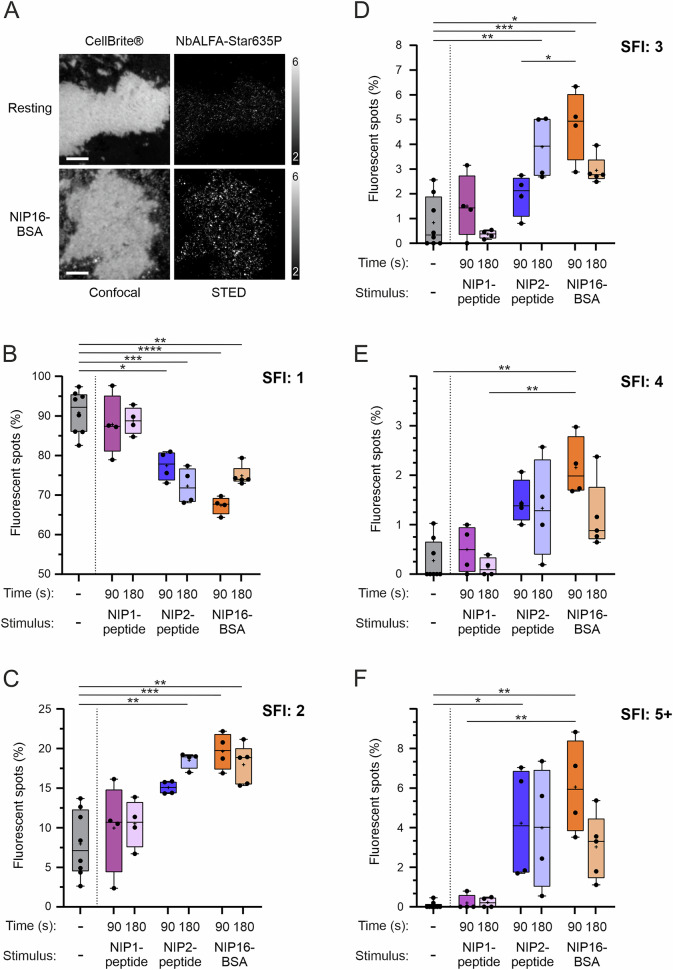
The degree of clustering determines the strength of BCR signaling. **A** Representative images of plasma membrane sheets from resting (upper row) or NIP16-BSA-stimulated (lower row) RHLKO cells expressing monovalent mIgG-BCRs. The membranes were stained with CellBrite® Green, and the BCRs were labeled with NbALFA FluoTag®-Q AbberiorStar635P. **B** Percentage of fluorescent spots representing monovalent mIgG-BCRs, which have a spot fluorescence intensity (SFI) that is equivalent to the intensity of one AbberiorStar635P molecule. The cells were either left untreated (42 membrane sheets from 8 replicates (42/8)) or stimulated for either 90 or 180 s with the indicated antigens. The numbers of membrane sheets and replicates were as follows: NIP1-peptide, 90 s: 27/4; 180 s: 42/4; NIP2-peptide, 90 s: 36/4; 180 s: 44/4; NIP16-BSA, 90 s: 44/4; 180 s: 51/4. **C**–**F** Same analysis as in (**B**) showing SFIs that are equivalent to two (**C**), three (**D**), four (**E**) or five or more (5+) BCRs per spot. Statistical analysis was performed via two-way ANOVA followed by Tukey’s multiple comparison test. ****P < 0.0001, ***P < 0.001, **P < 0.01, *P < 0.05. The whiskers in the graphs represent the minimum and maximum values

Quantitative information about the arrangement of BCRs in the plasma membrane and about the components of the BCR’s intracellular signaling machinery is scarce. Previously, we developed a quantitative STED microscopy-based approach to study B-cell surface proteins in patches of plasma membrane, so-called membrane sheets [27]. We refined our original protocol and adapted it to investigate monovalent and divalent mIgG-BCRs in resting and antigen-stimulated cells (Supplementary Fig. S9). To this end, the cells were either left untreated or stimulated for 90 or 180 seconds with antigens of distinct valences. The cells were subsequently washed, fixed and stained with an anti-ALFA nanobody (NbALFA) coupled to a single fluorophore molecule (Supplementary Fig. S10). To obtain reference signal intensities for single AbberiorStar635P-coupled NbALFA molecules, we quantified the fluorescence signals of unbound nanobodies in each imaging session (Supplementary Fig. S11A). Note that our heterodimeric BCRs only have a single ALFA tag per receptor, thus allowing for the quantification of BCRs per diffraction-limited spot when using STED imaging. We then prepared membrane sheets to quantify the fluorescence intensities of spots representing either resting or stimulated BCRs (Supplementary Fig. S11 and Fig. 5A). When monovalent BCRs in resting cells were analyzed, we found that more than 90% of the spot fluorescence intensities (SFIs) represented monomeric cell surface receptors (Fig. 5B, SFI: 1), which is in agreement with our previous results on mIgM-BCRs [27]. Stimulation of monovalent BCRs with the monovalent NIP1 peptide did not alter their distribution in the plasma membrane. However, when cells expressing monovalent BCRs were incubated with the divalent NIP2 peptide or with polyvalent NIP16-BSA, there were substantial decreases in the percentages of spots with fluorescence intensities that represented monomeric BCRs both after 90 s and after 180 s of stimulation (Fig. 5B). Accordingly, the percentages of fluorescent spots with fluorescence intensities corresponding to BCR dimers (SFI: 2, Fig. 5C), trimers (SFI: 3, Fig. 5D), tetramers (SFI: 4, Fig. 5E) or higher-order multimers (five or more (SFI: 5+ ), Fig. 5F) increased after stimulation, which is consistent with antigen-induced clustering of otherwise monomeric BCRs. Notably, cells treated with NIP16-BSA presented a clear decrease in the percentage of SFIs greater than 1 (i.e., nonmonomeric BCRs) between 90 and 180 s of stimulation. This phenomenon was not observed with the divalent NIP2 peptide and can be explained by the accelerated internalization of monovalent BCRs upon incubation with polyvalent but not divalent antigens (see Fig. 4). Since internalized BCRs cannot be detected in plasma membrane sheets, they ‘disappeared’ in our imaging analyses. In summary, the results of our quantitative superresolution imaging of monovalent BCRs in the plasma membrane suggest that there is a linear correlation between BCR signaling strength and internalization on the one hand and BCR cluster scale on the other hand. Furthermore, these results indicate that the accelerated internalization of monovalent BCRs requires the formation of large (5+) BCR clusters.

### The BCR cluster size needs to surpass a threshold to initiate Ca^2+^ signaling and internalization

**Fig. 6:**
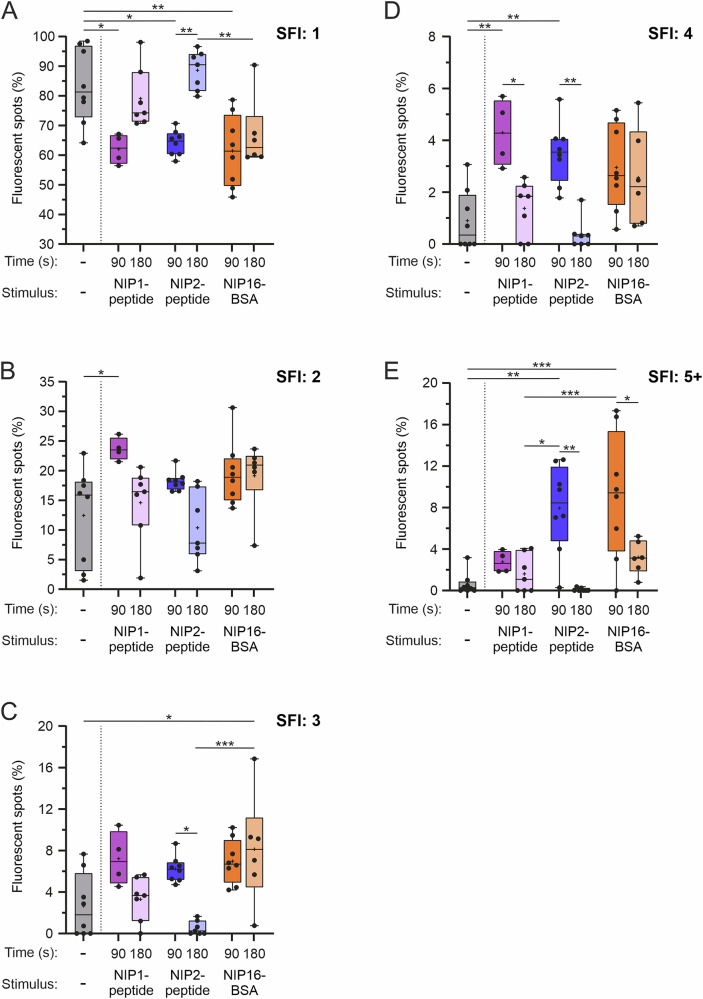
BCR signaling is initiated by 5+ clusters. RHLKO cells expressing divalent mIgG-BCRs were analyzed as shown in Fig. 5. Percentages of fluorescent spots that are equivalent to those of BCR monomers (**A**), dimers (**B**), trimers (**C**), tetramers (**D**) and pentamers and higher (**E**). The numbers of membrane sheets and replicates were as follows: resting: 55/8, NIP1-peptide, 90 s: 21/4; 180 s: 38/7; NIP2-peptide, 90 s: 49/8; 180 s: 41/7; NIP16-BSA, 90 s: 43/8; 180 s: 29/7. Statistical analysis was performed via two-way ANOVA followed by Tukey’s multiple comparison test. ****P < 0.0001, ***P < 0.001, **P < 0.01, *P < 0.05. The whiskers in the graphs represent the minimum and maximum values

While in B cells expressing monovalent BCRs, the relationship between BCR function and cluster size was relatively linear, the situation in cells expressing divalent BCRs was more complex. Somewhat unexpectedly, not only did divalent and polyvalent antigens cause detectable clustering of divalent mIgG-BCRs but also the signaling-inert monovalent NIP1 peptide, especially after 90 s of stimulation (Fig. 6A–E). However, the clustering of divalent BCRs by monovalent antigens was only transient, as it resolved after 180 s of stimulation and furthermore did not reach large cluster sizes (SFI: 5+). This was in stark contrast to stimulation with divalent and polyvalent antigens, both of which caused the formation of up to 10% 5+ clusters (Fig. 6E). As observed before, the percentages of BCR clusters decreased between 90 and 180 s of stimulation; however, this time also in the case of the dimeric antigen, which is in agreement with the accelerated internalization of divalent BCRs compared with their monovalent counterparts (see Fig. 4). Since the combination of divalent BCRs and divalent antigens resulted in a signaling strength comparable to that of monovalent BCRs and polyvalent antigens (see Fig. 2H), a particular signal intensity needs to be reached to induce accelerated BCR internalization (and, as a side effect, the ‘disappearance’ of clusters in our imaging analyses after 180 s of stimulation). The fact that the NIP1 peptide caused detectable formation of small clusters but did not induce BCR signaling further indicates that to activate the BCR signaling machinery, a cluster size threshold (probably 5+) must be surpassed. This was achieved by di- and polyvalent NIP antigens but not by the monovalent one. Together, our kinetics analyses of early changes in the arrangement of antigen-stimulated BCRs revealed that clustering of otherwise monomeric BCRs is the driving force for the activation of intracellular signaling and accelerated antigen internalization.

### Equipping BCRs with additional ITAMs causes cellular desensitization

**Fig. 7:**
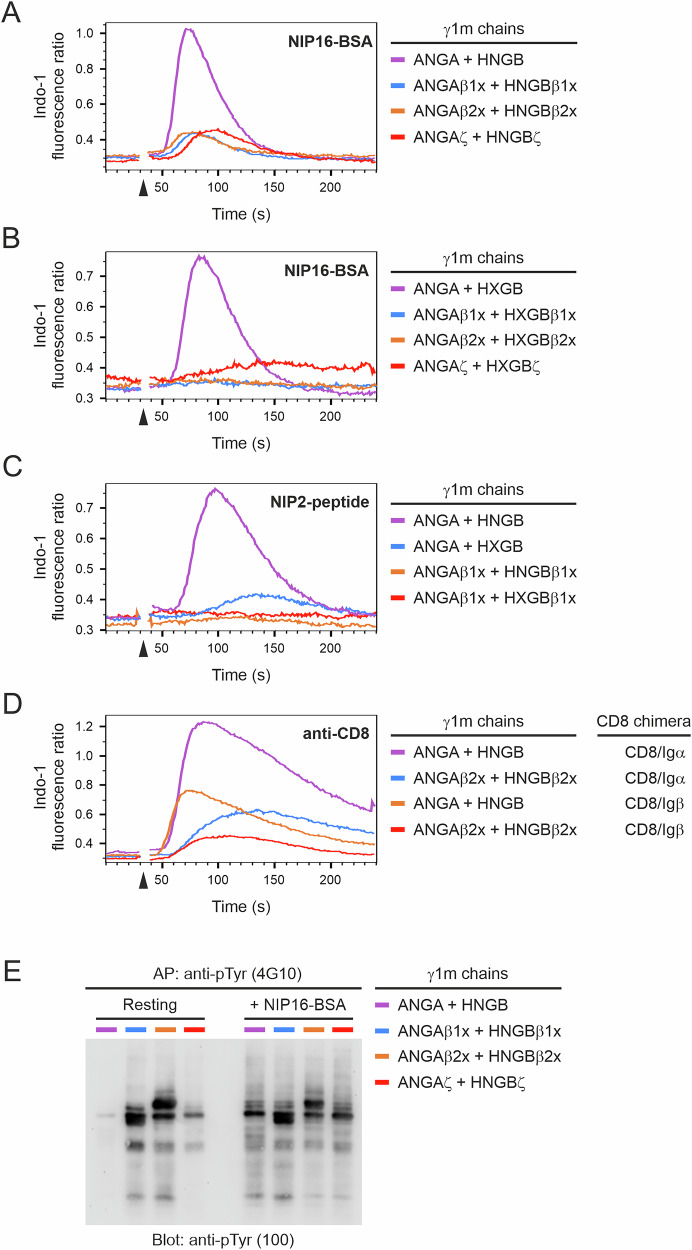
BCRs with additional ITAMs cause cellular desensitization. **A** Ca^2+^ mobilization analysis of RHLKO cells expressing divalent mIgG-BCRs with two (β1x), four (β2x) or six (ζ) additional ITAMs (for more details, see Supplementary Fig. S12). All BCRs were stimulated with NIP16-BSA. **B** Same experiment as in (**A**) with monovalent mIgG-BCRs. **C** Comparison of divalent and monovalent BCRs with or without two additional ITAMs upon stimulation with a divalent NIP peptide. **D** RHLKO cells expressing divalent mIgG-BCRs with or without four additional ITAMs and either CD8/Igα or CD8/Igβ were stimulated with an anti-CD8 antibody. **E** Tyrosine phosphorylation analysis of intracellular proteins from the cells shown in (**B**). The cells were either left untreated (resting) or stimulated for 3 min with NIP16-BSA at 37 °C. Subsequently, cell lysates were prepared, and tyrosine-phosphorylated proteins were purified with the anti-phospho-tyrosine antibody clone 4G10 and analyzed by immunoblotting with the anti-phospho-tyrosine antibody clone 100

Our results showed that clusters of five (or more) BCRs that are formed in the first few minutes after antigen contact appear to be signaling active. From an intracellular perspective, these clusters contain at least 10 copies of the ITAM signaling motif. Notably, a single, resting (monomeric) TCR already contains 10 ITAMs, which resembles the situation of a pentameric cluster of antigen-ligated BCRs. Thus, we sought to investigate how B and T cells cope with these different numbers of ITAM copies in their antigen receptors. To provide additional ITAMs to the mono- and divalent mIgG-BCRs, we fused either a single or double copy of the cytoplasmic domain of Igβ, or the cytoplasmic domain of the TCR ζ chain, to our γ1m A and B chains (see Supplementary Fig. S12 for details). This strategy resulted in the incorporation of two, four or six additional ITAM copies per BCR. All the BCR variants were expressed in similar amounts on the surface of the RHLKO cells (Supplementary Fig. S12). When stimulated with polyvalent NIP16-BSA, both di- and monovalent BCRs were severely compromised in their ability to induce Ca^2+^ mobilization, irrespective of the additional ITAM copy number (Fig. 7A, B). A similar effect was observed when we expressed and analyzed the mIgG-ζ chain chimeric BCR in the murine B-cell line WEHI-231 (Supplementary Fig. S13). Whereas divalent BCRs still showed a residual response in Ramos B cells, monovalent BCRs with as few as two additional ITAMs were nonresponsive. When stimulated with the divalent NIP2 peptide, the presence of two additional ITAM copies caused complete silencing of both mono- and divalent BCRs (Fig. 7C). To test whether the blunted Ca^2+^ responses were due to a malfunction of the modified BCRs or whether the cells’ intracellular signaling machinery in general was impaired, we undertook several experiments. First, we tested whether the internal Ca^2+^ stores of the cells were affected, by incubating the cells with thapsigargin, an inhibitor of the sarco/endoplasmic reticulum Ca^2+^ ATPase. However, the cellular Ca^2+^ stores were not significantly altered compared with those in control cells expressing mono- or divalent BCRs (Supplementary Fig. S14). Next, we equipped the cells with chimeric surface receptors containing the extracellular domain of CD8α and the intracellular domain of either Igα or Igβ (see Supplementary Fig. S15). These CD8 chimeras were stimulated with anti-CD8 antibodies to test their ability to initiate Ca^2+^ mobilization. Both chimeras were able to trigger Ca^2+^ responses similar to those seen by BCR activation in cells expressing mono- or divalent BCRs without additional ITAMs (Fig. 7D, purple and orange curves). However, in cells expressing BCRs with additional ITAM copies, the activity of the CD8 chimeras was strongly reduced (Fig. 7D, blue and red curves), indicating that the inhibition of signaling is not restricted to the modified BCRs but affects the cellular signaling machinery. This raised the possibility that BCRs with more than two ITAM copies are constitutively signaling active. Consistent with this notion, cells that expressed BCRs with additional ITAMs presented constitutive tyrosine phosphorylation of discrete protein bands in resting cells, the intensities of which were only moderately increased upon stimulation with polyvalent antigen (Fig. 7E). In contrast, cells expressing conventional divalent BCRs with only two ITAMs presented almost no constitutively tyrosine-phosphorylated proteins but a robust increase in tyrosine phosphorylation upon antigen stimulation (Fig. 7E, ANGA + HNGB). In additional experiments, we tested the phosphorylation status of the BCR transducer PTK Syk and that this enzyme is constitutively tyrosine-phosphorylated in B cells expressing BCRs with additional ITAM copies (Supplementary Fig. S15D), indicating constitutive Syk and, thus, BCR activation. In conclusion, lymphocytes seem to have adapted their intracellular signaling machinery to the unique numbers of ITAM copies in their respective antigen receptors. Increasing the number of ITAMs in a BCR probably mimics a constitutively clustered receptor, to which the signaling machinery in B cells adapts by desensitization.

## Discussion

The divalent core structure of Igs is an evolutionarily conserved trait. While the presence of two identical binding sites in a monomeric antibody results in an obvious gain in avidity, even single-domain binders can reach exquisite affinities, as evidenced by nanobodies and single-chain variable fragments [37, 38]. Moreover, the TCR naturally has only a single binding domain, the affinity of which is typically lower than that of (affinity-matured) antibodies but nonetheless is sufficient to allow for antigen recognition in the context of an immunological synapse [39]. Hence, nature itself has proven that monovalent antigen recognition is feasible, which raises the question of why all Igs have preserved their divalent core structure. Notably, each soluble Ig molecule (either monomeric or multimeric) has a cell surface-bound monomeric precursor isoform, which is expressed in the context of the BCR. Here, we showed that these mIg isoforms must be divalent to ensure proper function of the BCR. Thus, these isoforms may be responsible for the striking evolutionary conservation of the divalent Ig core structure. While we cannot study antibody production in cell line-based model systems, numerous reports have established a correlation between BCR signaling quality and antibody production in vivo. Our results indicate that the divalence of BCRs seems to be of particular importance for B cells to respond to low-valence antigens, which may be exemplified by small protein aggregates with only a few identical epitopes [40, 41]. Furthermore, the BCR divalence-dependent ability of B cells to respond to low-valence epitopes strongly enhances the immunogenicity of vaccines [36].

Surprisingly, the initial change of state that BCRs undergo to switch from resting, indolent to signaling-active receptors remains incompletely understood and thus has been a cause of disagreement in the field for a long period of time [21, 42]. The observations presented here support the concept of antigen-mediated cross-linking or clustering of otherwise monomeric BCR units as one mechanism to initiate intracellular events of signaling and accelerated receptor endocytosis. In this context, the function of extracellular multivalent antigens is analogous to that of intracellular adaptor proteins, which physically connect individual components that otherwise meet only very inefficiently by chance and thus remain largely inert. In this context, the BCR acts as a sensor of ligand valence. Accordingly, the monovalent NIP peptide in our experiments was unable to elicit BCR activation. Similar findings with monovalent NIP antigens were previously reported by others [43, 44]. In contrast, some groups have reported BCR activation by apparently monovalent NIP antigens [45, 46]. However, when we tested one of these apparently monovalent NIP antigens, it behaved like our divalent NIP peptide in Ca^2+^ mobilization assays (Supplementary Fig. S16). Nonetheless, we, too, observed the formation of small BCR clusters when divalent BCRs were stimulated with a monovalent NIP peptide. However, these clusters remained small and did not initiate signaling, indicating that signal initiation requires the formation of larger BCR clusters, i.e., pentamers and higher. Furthermore, the differences that we observed in BCR cluster size and numbers upon stimulation with divalent or polyvalent antigens were relatively small, indicating that B cells may sense rather subtle changes in BCR cluster scale, which are then translated into biologically meaningful cellular responses. In line with this notion, the cell surface organization of pathological BCRs with chronic signaling activities derived from chronic lymphocytic leukemia cells was only slightly skewed toward small clusters [27, 47].

Furthermore, experiments with PLCγ2 have shown that only a small fraction of the cellular pool of this enzyme is activated upon maximal BCR stimulation and that minute changes in the fraction of phosphorylated PLCγ2 are translated into a full Ca^2+^ response [48]. Thus, the dynamic range that is used by the BCR and its intracellular signaling machinery may actually be rather small. This assumption is supported by our finding that the incorporation of as few as two additional ITAMs into a BCR is sensed by a B cell, or more precisely by its intracellular signaling apparatus, and causes its desensitization, probably as a result of feedback mechanisms emanating from constitutive BCR signaling. This finding indicates the existence of a delicate balance of ITAMs and the signaling equipment that they control and that this balance has been adjusted throughout evolution in different types of lymphocytes. The TCR contains only one binding site and thus cannot be efficiently clustered by antigen (which, in the case of peptide/MHC complexes, is monovalent). Thus, the TCR may have incorporated 10 ITAMs into one monomeric unit to fulfill the needs for efficient activation of the T-cell signaling machinery [49].

The high-affinity Fc epsilon receptor I (FcεRI) is another member of the multiprotein immune receptor family that employs the ITAM signaling motif. A monomeric FcεRI contains three ITAMs and uses a monomeric (divalent) IgE antibody as antigen recognition device. Thus, when reduced to the copy numbers of antigen-binding domains and ITAMs, the FcεRI and BCRs are very similar. Importantly, signal initiation of the FcεRI relies on cross-linking by antigens, and low-valence antigens are sufficient to activate this receptor by forming small clusters [50, 51]. This finding indicates that divalent immune receptors with low numbers of ITAM copies that respond to soluble ligands share a common activation principle.

However, several laboratories have described the activation of BCRs by monomeric (and thus monovalent) protein antigens [45, 52–54]. Given that the antigens used were indeed pure monomers, there must be an additional activation mechanism for the BCR. In fact, some groups have provided evidence for conformational changes in BCRs after stimulation with membrane-embedded (i.e., partially immobilized) monovalent antigens [25, 28]. Thus, in addition to being crosslinked by antigens, BCR activation may also be achieved by conformational rearrangements in the receptor complex, at least in the case of certain antigens.

More recently, it has been demonstrated that BCRs on resting cells are not evenly distributed in the plasma membrane. Three-dimensional microscopic analysis of cell surface mIgM in whole cells revealed the existence of topographical areas in which the local concentration of BCRs was either higher or lower than average [55]. The highest density was found at the tips of cellular protrusions to which the BCR was actively transported. Another study confirmed the existence of areas with locally enriched mIgM-BCRs, albeit without information about the cell surface regions in which these areas reside [46]. Unfortunately, both studies classified these areas as ‘BCR clusters’, even though the resolution limit either was too low to make that claim [55], or the authors themselves concluded that BCRs in these areas were too far separated to be physically connected and thus, in fact, represented monomeric units [46]. We propose to reserve the term ‘BCR cluster’ only for those receptors that, in all probability, are physically connected either by antigen or by homotypic interactions such as autoaggregation. Nonetheless, locally confined areas of preconcentrated BCRs in certain topographical regions of the cell surface may support the receptors' function by increasing the probability of true cluster formation either by antigen contact or by stochastic collisions, the latter of which may be relevant for the tonic survival activity of the BCR.

In summary, the findings presented herein improve our understanding of the molecular principles that underlie the activation and regulation of ITAM-bearing immunoreceptors. This topic has become increasingly important since manmade, synthetic ITAM-based receptors have found their way into clinical applications in the context of cellular immunotherapies.

## Materials and methods

### Cells and expression vectors

A variant of the human Burkitt lymphoma B-cell line Ramos that lacks expression of endogenous immunoglobulin heavy and light chains (RHLKO) was provided by Dr. Michael Reth (Freiburg, Germany). All cells were maintained in RPMI 1640 + GlutaMAX (Biochrom, Berlin, Germany) supplemented with 10% heat-inactivated FCS and antibiotics. All cDNAs encoding immunoglobulin heavy and light chains were synthesized by Eurofins Genomics (Ebersberg, Germany) and ligated into the retroviral expression vector pMSCVpuro (Clontech, Takara Bio Inc., Kyoto, Japan). Retroviral particles were produced using the packaging cell line Plat-E. The vesicular stomatitis virus (VSV) glycoprotein was used to pseudotype MMLV particles for human cell transduction. Transduced cells were selected in the presence of 2 µg/ml puromycin for 5‒7 days, followed by expression analysis via flow cytometry and Western blotting. When necessary, the transduced cells were sorted for purity using a BD FACSAria II.

### Flow cytometry

The cell surface expression of the immunoglobulin chains was analyzed via flow cytometry using the following reagents: FluoTag®-X2 anti-ALFA AlexaFluor 647 (Nanotag Biotechnologies, Göttingen, Germany), Brilliant Violet 421™ anti-HA.11 Epitope tag antibody (BioLegend, San Diego, CA, USA) and NP-PE (Biosearch Technologies, Petaluma, CA, USA). For each sample, 1 × 10^6^ cells were stained for 30 min on ice in the dark, followed by three wash steps. For the staining of intracellular proteins, the cells were fixed in Cytofix buffer (BD Biosciences) for 20 min on ice, followed by incubation in Perm/Wash Buffer I (BD) for another 20 min at 22 °C. Staining was performed in Perm/Wash buffer I using anti-phospho-CD79A-AF647 (Y182, Cell Signaling Technology, Danvers, MA, USA) and anti-phospho-Syk-APC (Y348, Invitrogen) antibodies. Finally, the cells were washed three times for 10 min each in Perm/Wash buffer I. All flow cytometry data were acquired on FACSCelesta or LSRII flow cytometers (Becton Dickinson, Franklin Lakes, NJ, USA) and analyzed via FlowJo 10.8 software. Imaging flow cytometry of TFEB was performed as described previously [18]. In brief, the cells were fixed with CytoFix buffer for 20 min at 4 °C, washed with PBS and resuspended in 200 μl of PBS containing 0.1% Triton X-100, 2% FCS and a primary antibody against human TFEB (clone D2O7D, CST, Danvers, MA, USA). After incubation at 4 °C for 30 min and washing with PBS, the cells were stained with an AlexaFluor-647-conjugated secondary anti-rabbit antibody (Abcam) and incubated at 4 °C for 30 min. After washing with PBS, the nuclei were stained with 50 µl of PBS containing 1 µg/ml DAPI (Thermo Fisher). For each sample, at least 2 × 10^4^ single, focused cells (as determined via the area-to-aspect ratio and GradientRMS, respectively) were recorded via an ImageStreamX MkII imaging flow cytometer (Luminex). Apoptotic cells were excluded by means of their nuclear morphology (‘Apoptosis Wizard’ algorithm, DAPI staining). The nuclear localization of TFEB was assessed via the ‘Nuclear Translocation Wizard’ algorithm, which is based on channels 11 (TFEB/AF647) and 7 (nucleus/DAPI). The nucleus was defined via the mask ‘Dilate(Object(M05,Ch05,Tight),1)’. Events with a ‘SimilarityDilate’ colocalization score >1.0 were considered cells with nuclear localization of TFEB.

### Measurement of intracellular free Ca^2+^

The day before the measurement, ~1 × 10^6^ cells were seeded in a 10 cm culture dish. The next day, the cells were gently mixed for 30 min at 30 °C with 1 µM Indo-1-AM (Invitrogen, Thermo Fisher Scientific, Waltham, MA, USA) in RPMI containing 10% fetal bovine serum and 0.015% Pluronic-F-127 (Invitrogen). The cells were subsequently washed twice and resuspended in Krebs-Ringer solution composed of 10 mM HEPES (pH 7.0), 140 mM NaCl, 10 mM glucose, 4 mM KCl, 1 mM MgCl_2_ and 1 mM CaCl_2_. After 30 min of rest prior to measurement, the fluorescence ratio of Ca^2+^-bound Indo-1 (405 nm) to Ca^2+^-unbound Indo-1 (530 nm) was monitored on a FACSCelesta cytometer. The basal Indo-1-AM ratio was monitored for 30 seconds, followed by stimulation with the indicated reagents. Goat anti-human F(ab′)_2_ fragments against human IgG or κ were obtained from Southern Biotech (Birmingham, AL, USA). NIP-16 and NIP-24-BSA were purchased from Biosearch Technologies (Petaluma, CA, USA). NIP-containing peptides were synthesized by CASLO ApS (Lyngby, Denmark) and had the sequences (Biotin)-KSK(NIP)GESKG (NIP1-peptide) and (Biotin)-KSK(NIP)GESK(NIP)G (NIP2-peptide), respectively. Anti-human CD8 (clone MEM-31) was a gift from Dr. Vaclav Horejsi (Prague, Czech Republic). Purified streptavidin was obtained from Jackson ImmunoResearch (West Grove, PA, USA). The data were analyzed via FlowJo (FlowJo LLC, Ashland, OR, USA), Microsoft Excel and GraphPad Prism.

### Antigen endocytosis assay

To analyze the internalization of BCR-bound antigens, NIP-specific B cells were incubated either with biotinylated NIP peptides (10 ng/ml) or with biotinylated NIP16-BSA (500 ng/ml) in PBS for 10 min on ice to allow for antigen binding. The cells were subsequently pelleted and washed with PBS. The cell solutions were split into 6 tubes, each of which was incubated at 37 °C for the indicated period of time (unstimulated cells (time point 0) were kept on ice). The cells were subsequently stained with streptavidin-APC (BD Biosciences) for 10 min on ice, followed by another wash step with ice-cold PBS. Finally, the cells were analyzed on an LSRII flow cytometer. The MFIs of unstimulated cells were set to represent 100% bound antigen, and all other MFIs were normalized accordingly.

### Biochemical assays, antibodies and reagents

Preparation of cellular lysates in 1% NP40-containing lysis buffer for affinity purification and western blot analyses was performed as previously described [9]. In brief, the cells were lysed with lysis buffer composed of 50 mM Tris-HCl (pH 7.8), 137 mM NaCl, 0.5 mM EDTA, 1 mM sodium orthovanadate, 10% (v/v) glycerol, 1% (v/v) NP40 and a protease inhibitor cocktail containing AEBSF, Aprotinin, Bestatin, E-64, Leupeptin and EDTA (Sigma Aldrich, #P2714). Lysis was performed on ice for 10 min, and the cell debris was pelleted at 20,000 × *g* at 4 °C for 10 min. The supernatant containing the cell lysate was mixed with reducing SDS‒PAGE sample buffer, boiled at 95 °C for 5 min and analyzed by SDS‒PAGE and immunoblotting. For affinity purification of proteins from B cells, cell lysates were prepared as described above from 3 × 10^7^ Ramos cells and incubated with either 2 µg of monoclonal antibody 4G10 (Merck Millipore) or 2 µg of biotinylated anti-ALFA sdAb (Nanotag, Göttingen, Germany). Purification was performed via either Protein A/G PLUS agarose (Santa Cruz Biotechnology, Santa Cruz, CA, USA) or streptavidin-Sepharose (GE Healthcare, Chicago, IL, USA) under gentle rotation at 4 °C for 2 h. All affinity-purified samples were washed three times with lysis buffer before they were boiled in Laemmli buffer at 95 °C for 5 min and subjected to SDS‒PAGE and immunoblotting. The phospho-tyrosine antibody (clone 100) was from Cell Signaling Technology (Danvers, MA, USA), and the monoclonal anti-Syk antibody (clone 4D10) was from Santa Cruz Biotechnology (Santa Cruz, CA, USA). The immunoblot images were processed via Photoshop CS4 and Corel Draw software. The band intensities were quantified with LabImage 1D software (Kapelan Bio-Imaging, Leipzig, Germany).

### Generation of plasma membrane sheets

As a quality control measure, prior to each round of membrane sheet generation, the cell viability and response to antigen stimulation were assessed via Ca^2+^ mobilization assays, as described in the methods section. The plasma membrane sheets were prepared via a combination of methods described by Gomes de Castro et al. [27] and Erlendsson et al. [56], with a few modifications. The newly developed technique is illustrated in Supplementary Fig. S9. Briefly, ~3 × 10^6^ cells per condition were freshly taken from the cell culture, transferred to a low-bind 1.5 ml tube (Sigma‒Aldrich, cat. no. Z666548-250EA), pelleted at 300 × *g* for 5 min, and washed once in PBS (Sigma, cat. no. D8537) at room temperature (RT). The cells were subsequently pelleted again and resuspended in Krebs-Ringer solution (120 mM NaCl, 5 mM KCl, 2 mM CaCl_2_, 1 mM MgCl_2_, 25 mM NaHCO_3_, and 5.5 mM D-glucose). Cell stimulation was performed at this step in Krebs-Ringer solution by adding the indicated antigens. The cells were immediately fixed either 90 s or 180 s after the addition of ligand by adding an equal volume of fixation solution (8% paraformaldehyde, 0.2% glutaraldehyde in PBS; i.e., final concentrations were 4% paraformaldehyde, 0.1% glutaraldehyde) and incubating at RT for 30 min. The fixation solution was removed after centrifugation, and the cells were washed three times with quenching buffer (0.1 M glycine in PBS). For staining, the cells were incubated with 10 nM FluoTag®-Q AbberiorStar635P anti-ALFA (NanoTag Biotechnologies, Göttingen, Germany; cat. no. N1502-Ab635P-L) for 1 h at RT, followed by three washing steps with PBS. After staining, the cells were postfixed with 4% paraformaldehyde and 0.1% glutaraldehyde in PBS for 15 min at RT, followed by three rounds of 5-minute washing steps with quenching buffer. The cells were then stained with CellBrite™ Green membrane dye (Biotium Inc., Freemont, CA, USA, cat. no. 30021) diluted 1:500 in PBS for 15 min at RT and washed three times with PBS. The cells were transferred onto poly-L-lysine (PLL)-coated coverslips (ø 18 mm) placed in 12-well plates (Thermo Scientific™, cat. no. 150200) and centrifuged at 300 × *g* for 5 min at RT to allow the cells to sediment onto the coverslips. A second clean coverslip was placed on top of the cells, creating a coverslip-cell-coverslip “sandwich”. A flat plunger from a 10 ml syringe was used to gently put pressure onto the coverslip while the top coverslip was rotated clockwise, thus causing cell disruption and flattening of the cell membranes on the coverslips. Finally, the coverslips were mounted in Mowiol mounting media (12 ml 0.2 M Tris buffer pH 8.5, 6 ml ddH_2_O, 6 g glycerol, 2.4 g Mowiol, Carl Roth, cat. no. 0713.2).

### STED imaging

Images were acquired via an inverted Abberior STED Expert line microscope (Abberior Instruments, Göttingen, Germany) equipped with a UPLSAPO 100×1.4 NA oil immersion objective (Olympus). To ensure comparability of the data for quantitative analyses, the microscope settings were kept constant throughout all the experiments. The excitation lasers at 488 nm and 640 nm were set at a power of 1 μW, and the 775 nm depletion laser was set at 5 mW, which was measured at the sample. The laser power was measured via a microscope slide power sensor with a power and energy meter interface (S170C, Thorlabs; PM100USB, Thorlabs). The imaging settings were as follows: the pixel size was set to 20 nm with a dwell time of 10 μs and signal accumulation over 2 lines. Both confocal and STED images were collected from the 640 nm channel, whereas only confocal images were acquired from the 488 nm channel.

### Membrane sheet image analyses

All data analyses were performed via a custom-written GUI and scripts in MATLAB 2017a. Peak detection: For each acquired STED image $a$, regions of interest (ROIs) were manually selected to contain intact membrane patches and exclude high-intensity contamination (giving a binary mask $m$). To remove-of-focus signals and membrane autofluorescence, the images were high-pass filtered by subtracting a Gaussian-filtered version of the image excluding regions outside the selected ROIs:

$${a}_{{\mbox{h}}}=a\cdot m-\frac{{gaus}{s}_{\sigma }\left(a\cdot m\right)}{{gaus}{s}_{\sigma }\left(m\right)},$$

where ${gaus}{s}_{\sigma }$ is a 2D Gaussian filter with standard deviation σ = 8 pixels. This method constrains the filter inside the masked area and prevents high-intensity signals outside of the mask from influencing the subtracted background. In the resulting high-pass images ${a}_{{\mbox{h}}}$, local intensity maxima above a noise threshold were detected with subpixel accuracy, generating an initial list of $N$ peak center coordinates $\left({x}_{n},\,{y}_{n}\right)$.

Peak amplitude estimation by two-pass sparse deconvolution: Due to possible overlaps between nearby peaks, each pixel intensity inside the mask was modeled as a sum of intensity contributions from individual peak Gaussian point spread functions (PSFs):

$${a}_{{\mbox{fit}}}(x,y) = 	{\sum}_{n=1}^{N}\frac{1}{2}{A}_{n}\left({{{\rm{erf}}}}\left(\frac{{x}_{n}-x+1/2}{s}\right)-{{{\rm{erf}}}}\left(\frac{{x}_{n}-x-1/2}{s}\right)\right)\\ 	 \cdot \left({{{\rm{erf}}}}\left(\frac{{y}_{n}-y+1/2}{s}\right)-{{{\rm{erf}}}}\left(\frac{{y}_{n}-y-1/2}{s}\right)\right),$$

where ${A}_{n}$ represents the amplitude of an individual peak, $s$ represents the Gaussian PSF standard deviation, which is linearly related to the PSF’s full width at half maximum (FWHM), and erf() represents the error function used to calculate the pixel intensity integral. All valid pixel positions $(x,y)$ thus form an overdetermined system of linear equations with $N$ unknown amplitudes, solved by least-squares fit. Note that the PSF is assumed to be constant across all images.

As the overlap of multiple close-proximity peaks could make some of them nondetectable by the initial local maximum algorithm, a second pass of the above method was applied by first subtracting the modeled image ${a}_{{\mbox{fit}}}$ from ${a}_{{\mbox{h}}}$ (revealing unaccounted interpeak intensity), performing local maximum detection again on the difference image and fitting the amplitudes a second time using the original ${a}_{{\mbox{h}}}$ but with the combined set of peak candidates from the first and second passes.

Estimation of the monomer amplitude: Assuming a linear relationship between the total peak amplitude $A$ and the number of underlying labeled receptors r, $A={r\cdot a}_{0}$, the monomer amplitude ${a}_{0}$ was fit for the peak amplitudes ${A}_{n}$ by minimizing the cost function

$${{\mbox{cost}}}\left({a}_{o}\right)={\sum}_{n=1}^{N}{\min }_{r=1\ldots R}\left(\left|{A}_{n}-r\cdot {a}_{0}\right|\right)$$

represents the sum of the distances of all measured amplitudes to their respective nearest multiple monomer amplitudes (choosing from a total of ${R}$ possible receptor numbers).

Estimation of receptor number distribution: The number of receptors associated with each peak was estimated probabilistically. With the fitted monomer amplitude ${a}_{0}$, the peak amplitude ${A}_{n}$ was attributed to all receptor numbers $r=1$ to $R$ according to a Gaussian density ${w}_{n,r}$ centered at the expected value $r\cdot {a}_{0}$ and a standard deviation equal to half of the monomer intensity quanta ${a}_{0}$:

$${w}_{n,r}=\exp \left(-{\left(\frac{\left|{A}_{n}-r\cdot {a}_{0}\right|}{{a}_{0}/2}\right)}^{2}\right).$$

The overall receptor number distribution ${I}_{r}$ is then formed by the sum of these weights, normalized per peak:

$${I}_{r}=\frac{1}{N}{\sum}_{n=1}^{N}\frac{{w}_{n,r}}{{\sum }_{k=1}^{R}{w}_{n,k}}.$$

To display the relative contribution of individual receptor numbers to the total fluorescence amplitude, the receptor number distribution was then simply multiplied by its respective amplitude:

$${I}_{r}^{{\mbox{rel}}}={I}_{r}\cdot r\cdot {a}_{0}.$$

## Supplementary information

Supplementary information

unprocessed images
