# Supplementary material for: Immunoglobulin divalence promotes B-cell antigen receptor cluster scale-dependent functions
Source: Cell Mol Immunol. 2025 Aug 6;22(9):1093–108. doi: 10.1038/s41423-025-01327-1 (PMC12398502; doi:10.1038/s41423-025-01327-1)
Supplement: Supplementary file 1 — Supplementary information [file 41423_2025_1327_MOESM1_ESM.pdf]

Supplementary Material for

Immunoglobulin divalence promotes B cell antigen receptor cluster scale-dependent functions

Erdem Yilmaz, Amir Rahimi *et al.*

Corresponding authors: nengels@gwdg.de, fopazo@gwdg.de

This PDF file includes: Unprocessed western blots shown in Figures 1, 7 and S14

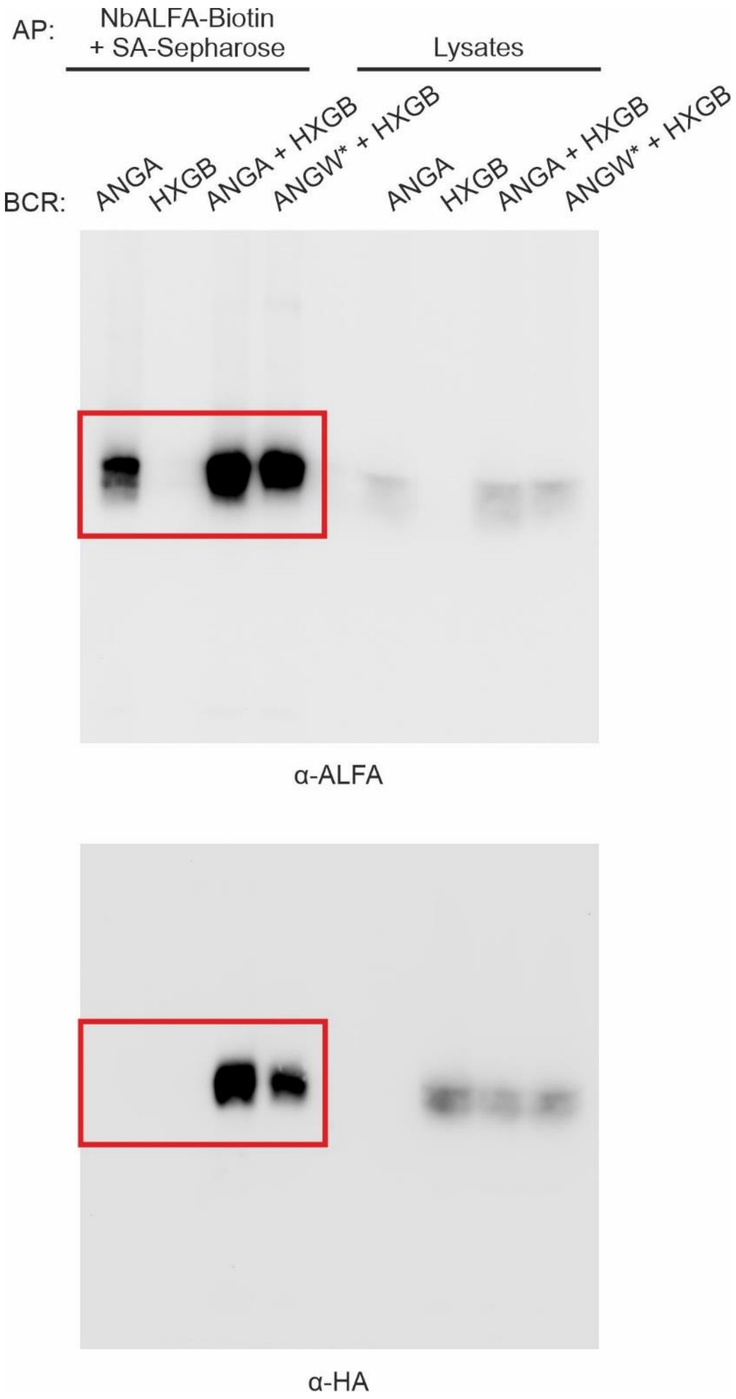

Uncropped western blots shown in Figure 1E. Cropped regions are indicated in red.

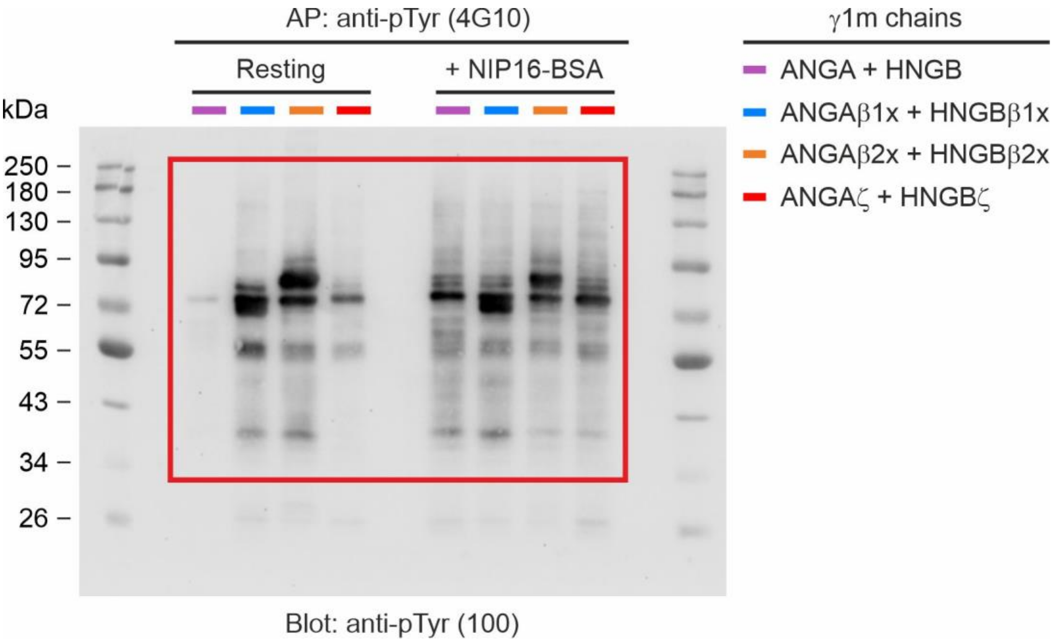

Uncropped western blot shown in Figure 7E. Cropped region is indicated in red.

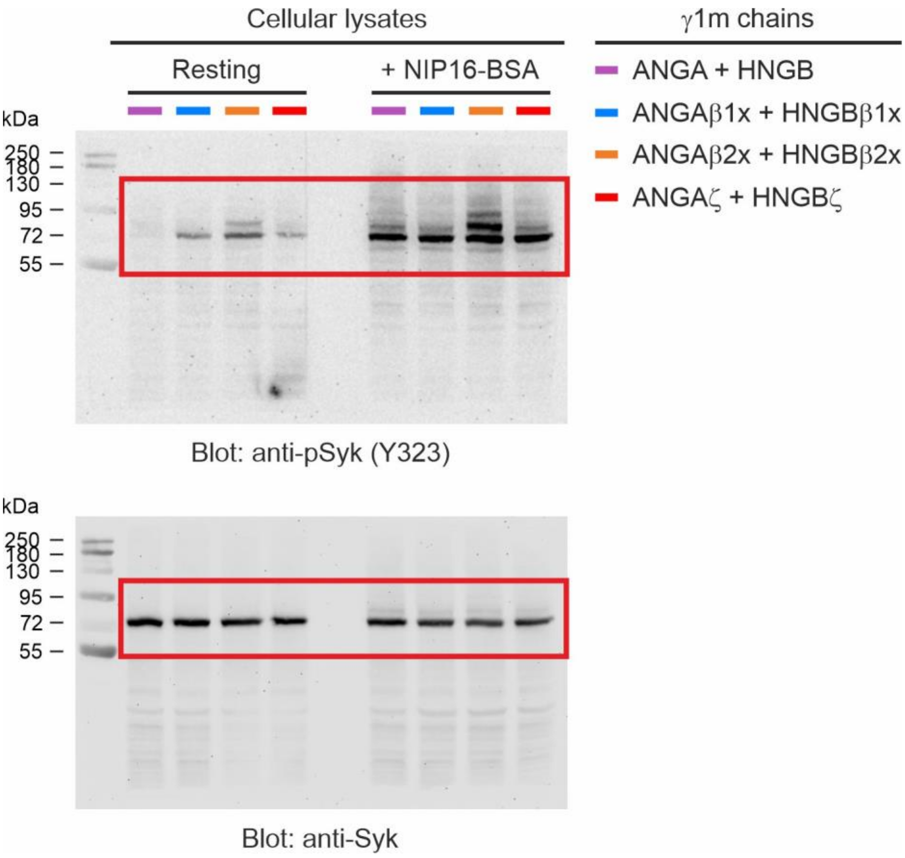

Uncropped western blots shown in Figure S15D. Cropped regions are indicated in red.
