# Supplementary material for: Immunoglobulin divalence promotes B-cell antigen receptor cluster scale-dependent functions
Source: Cell Mol Immunol. 2025 Aug 6;22(9):1093–108. doi: 10.1038/s41423-025-01327-1 (PMC12398502; doi:10.1038/s41423-025-01327-1)
Supplement: Supplementary file 2 — unprocessed images [file 41423_2025_1327_MOESM2_ESM.pdf]

Supplementary Figures for

Immunoglobulin divalence promotes B cell antigen receptor cluster scale-dependent functions

Erdem Yilmaz, Amirmohammad Rahimi *et al.*

Corresponding authors: nengels@gwdg.de, fopazo@gwdg.de

This PDF file includes: Figures S1 to S16

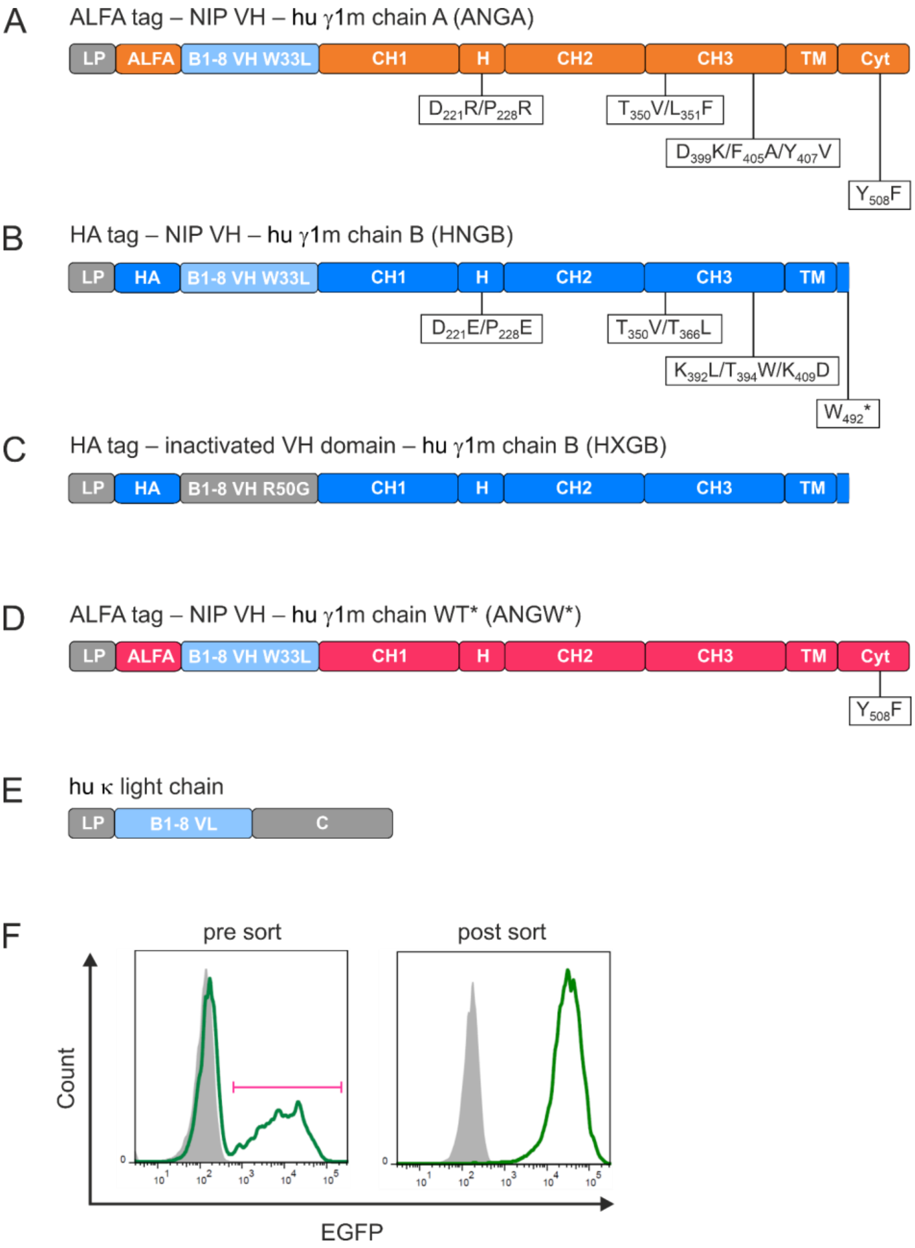

Figure S1. Generation of a heterodimeric mIgG-BCR. (**A**-**E**) Schematic illustrations of the cDNAs used to express homo- and heterodimeric human mIgG1. The modified γ1m A chain is shown in amber (**A**), the B chains in blue (**B**-**C**). A control chain that did not contain any amino acid substitution in the extracellular domains but a single Y-to-F replacement in the intracellular ITT motif (and hence is designated WT*) is shown in watermelon (**D**). (**E**) Schematic depiction of the used light chain. (**F**) FACS histograms of RHLKO cells that were retrovirally transduced to express a hybrid light chain consisting of the B1-8 VL domain and the human κ C domain. IRES-driven co-expression of EGFP was used as a surrogate marker to identify and sort LC-expressing cells.

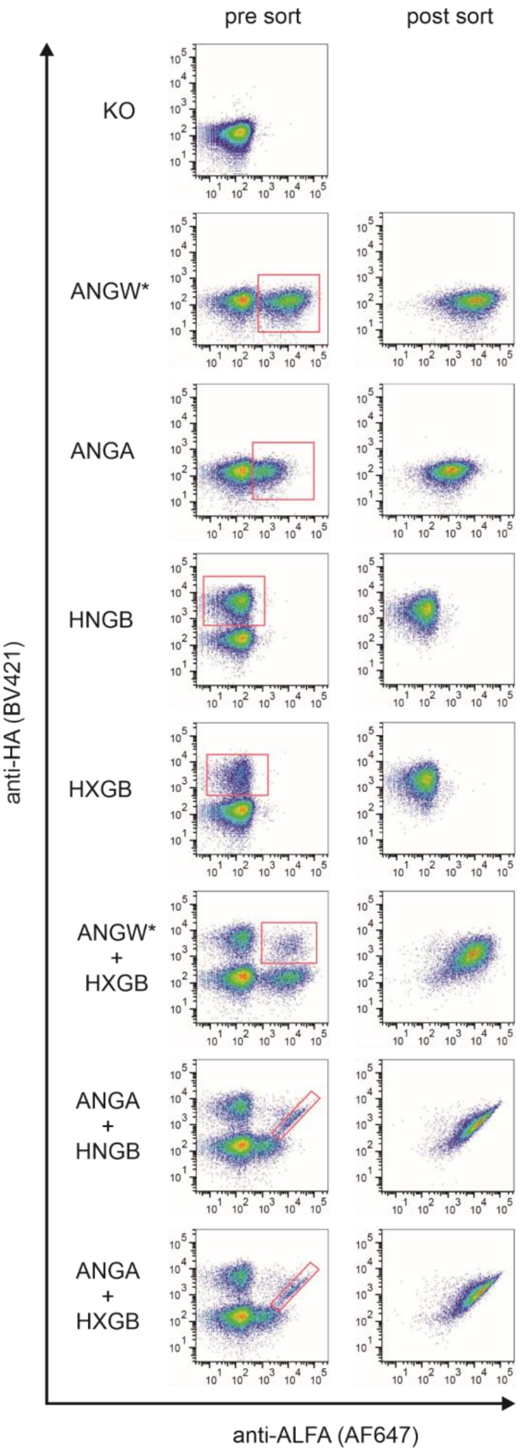

MFIs post sort

	ALFA (Chain A)	HA (Chain B)
ANGW*	13,500	140
ANGA	1,610	162
HNGB	118	2,540
HXGB	119	2,360
ANGW* + HXGB	9,430	1,460
ANGA + HNGB	12,900	1,310
ANGA + HXGB	14,300	1,440

Figure S2. Surface expression of homo- and heterodimeric mIgG-BCRs. Density plots of RHLKO/κLC cells that were retrovirally transduced to express the indicated γ1m HCs. Left column shows cells after puromycin selection. The gates that were applied for sorting are shown in purple. Right column shows cells after sorting. Mean fluorescence intensities (MFIs) of A and B chains after sorting are given on the right.

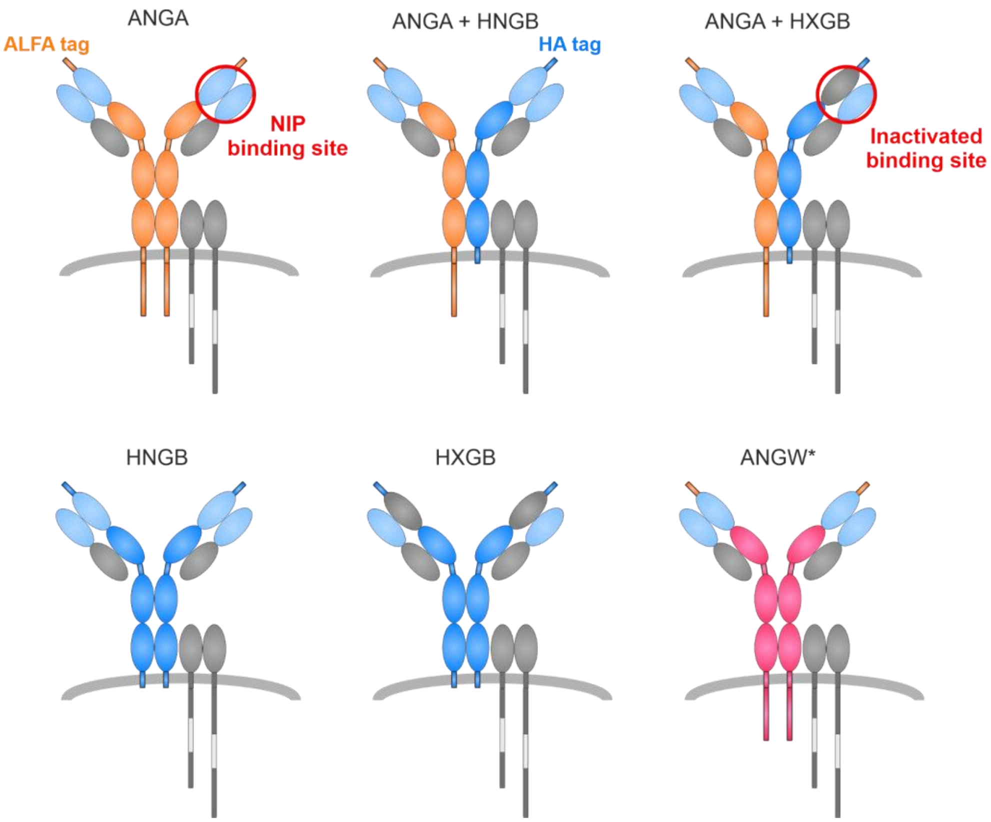

Figure S3. Schematic illustrations of the various homo- and heterodimeric mIgG-BCRs. Color code for heavy and light chains is the same as in Figure S1.

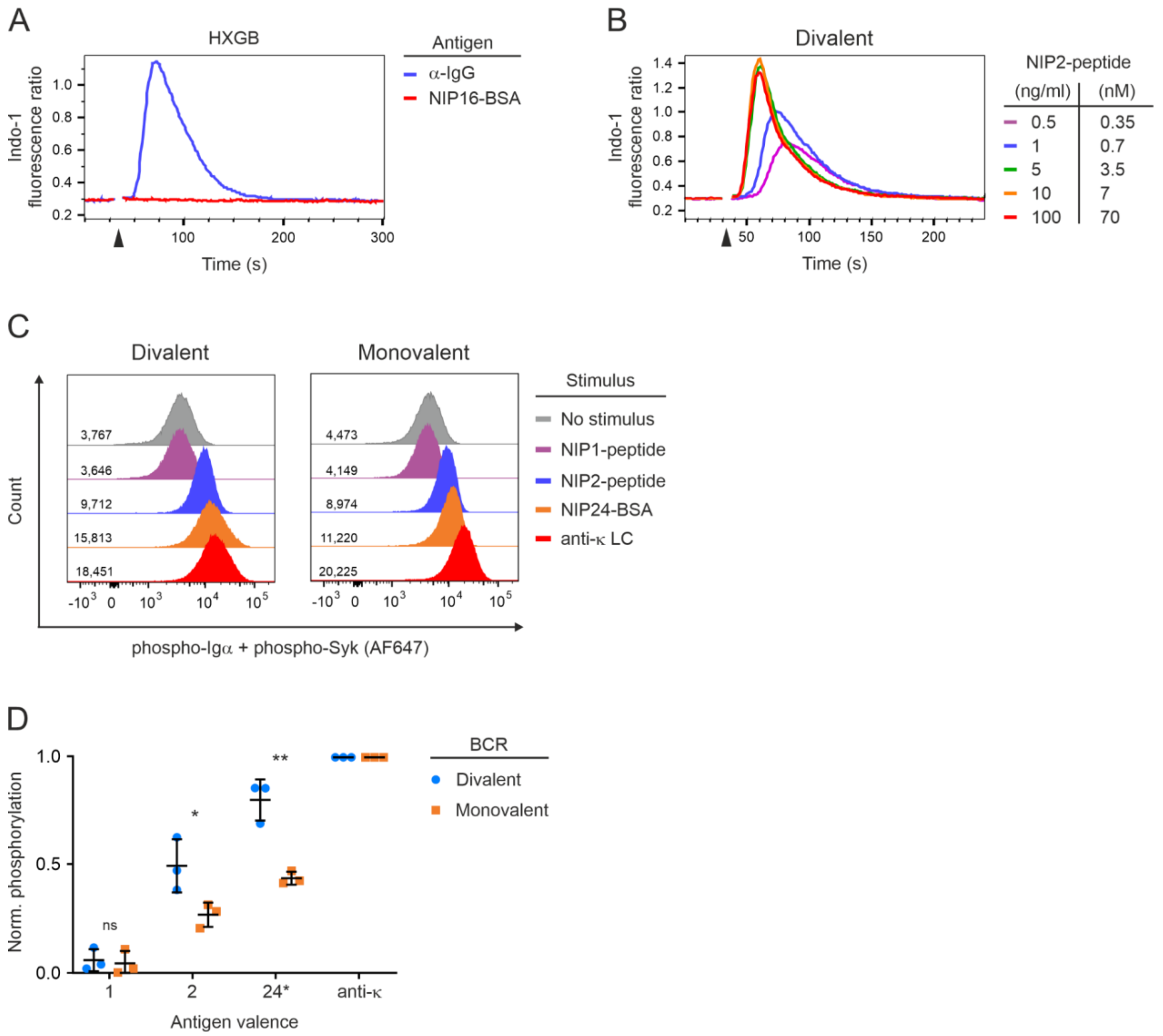

Figure S4. Antigen-induced signaling in RHLKO cells expressing mono- or divalent mIgG-BCRs. (**A**) Ca^{2+} signaling of cells expressing an mIgG-BCR containing a homodimer of HXGB chains on stimulation with either anti-IgG $F(ab')_2$ fragments (blue curve) or NIP16-BSA (red curve). (**B**) Titration of the NIP2-peptide in RHLKO cells expressing a divalent NIP-reactive BCR. All subsequent stimulation experiments with NIP-peptides were carried out with 10 ng peptide per ml (orange curve). (**C**) Combined analysis of tyrosine phosphorylation of Igα (Y182) and Syk (Y348) in cells expressing either di- or monovalent mIgG-BCRs. Cells were stimulated with the indicated reagents for three minutes at 37°C and tyrosine-phosphorylation was tested by intracellular phos-flow. Numbers show MFIs of AF647. (**D**) Statistical analysis of experiment shown in (C). Error bars represent mean ± SD of three independent experiments. Statistical significance was tested in GraphPad Prism using multiple t-tests. $*p \leq 0.05$; $**p \leq 0.01$; $***p \leq 0.001$; $****p \leq 0.0001$; ns, not significant.

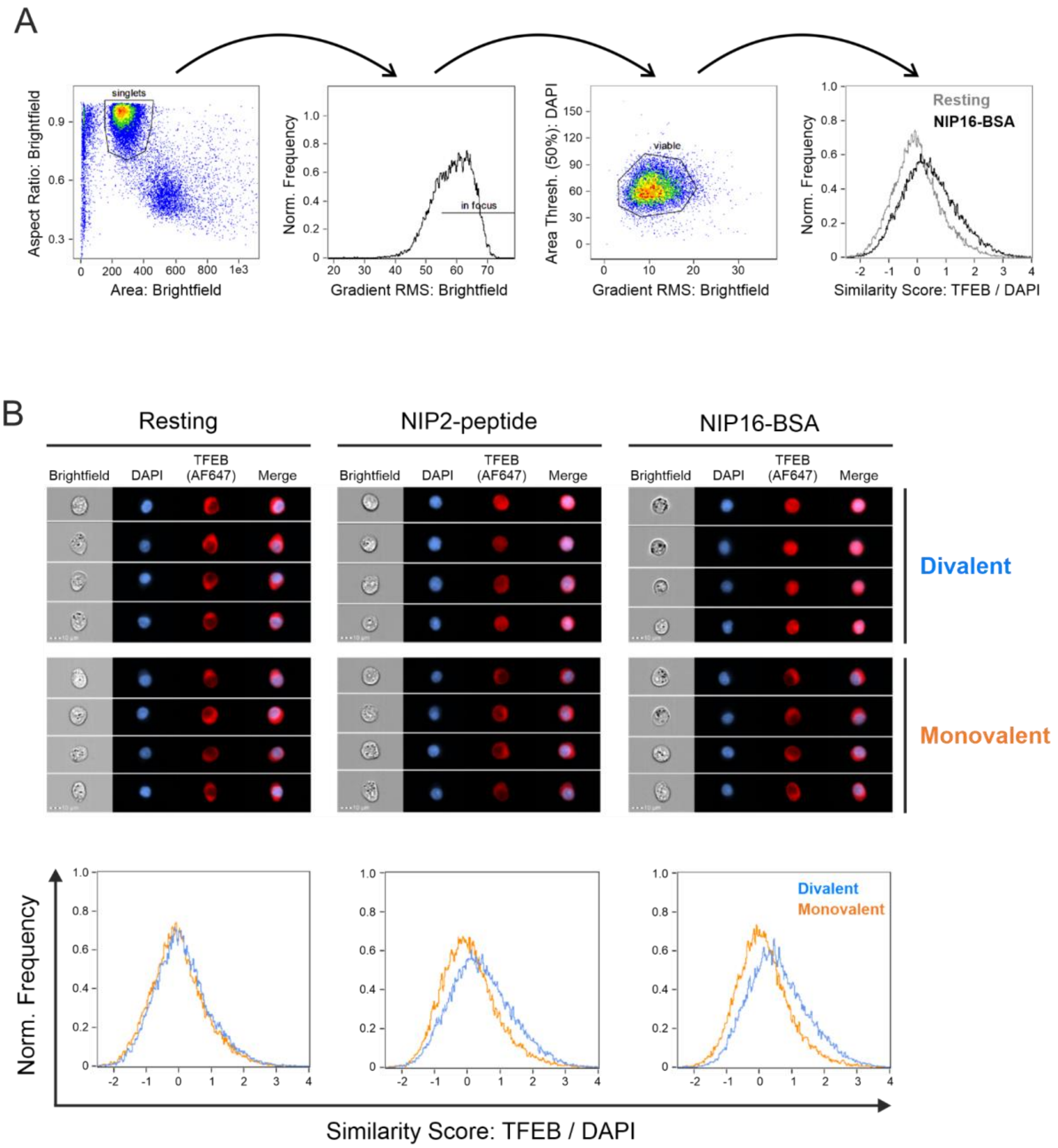

Figure S5. BCR-induced nuclear translocation of TFEB requires immunoglobulin divalence. (**A**) Imaging flow cytometry gating strategy to identify single, focused, viable cells. (**B**) Analysis of the sub-cellular distribution of TFEB in resting and antigen-stimulated cells expressing either divalent or monovalent mIgG-BCRs.

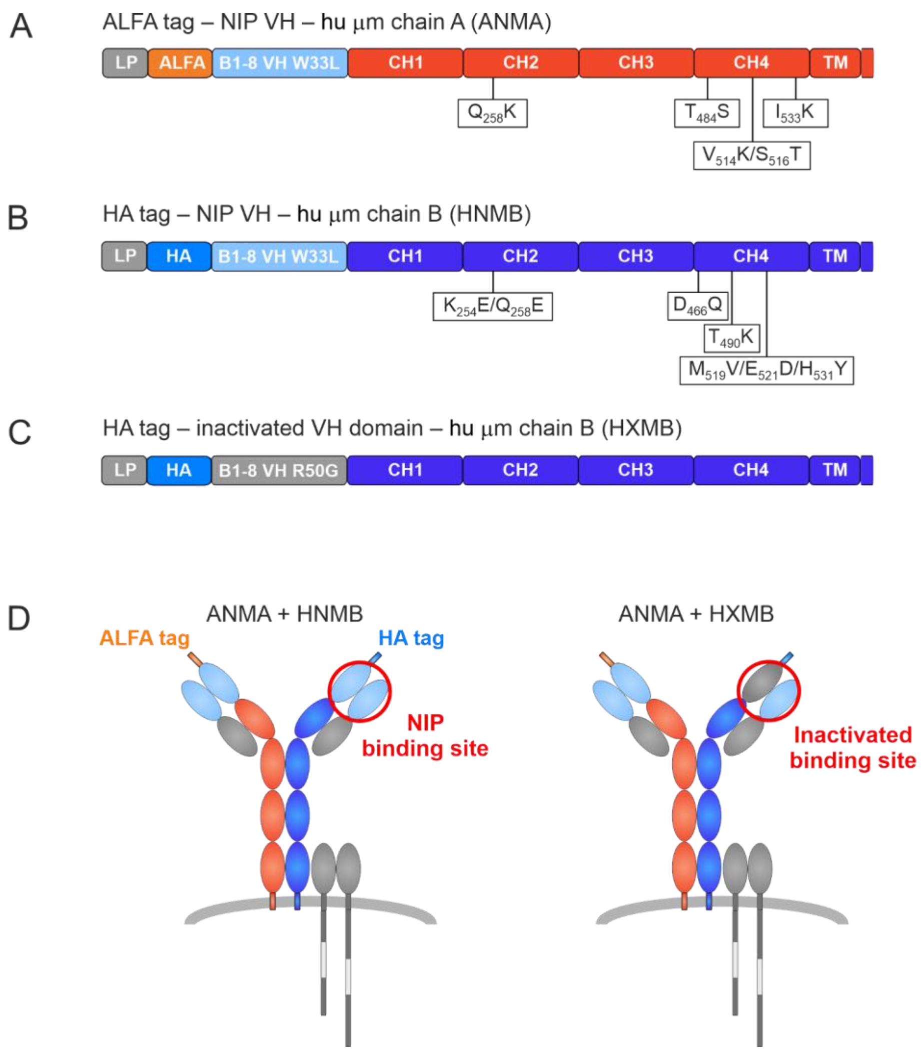

Figures S6. Generation of a heterodimeric mIgM-BCR. Schematic illustrations of the cDNAs used to express homo- and heterodimeric human mIgM. The modified μm A chain is shown in red (**A**), the B chains are shown in dark blue (**B**-**C**). (**D**) Schematic illustrations of divalent and monovalent, NIP-reactive mIgM-BCRs.

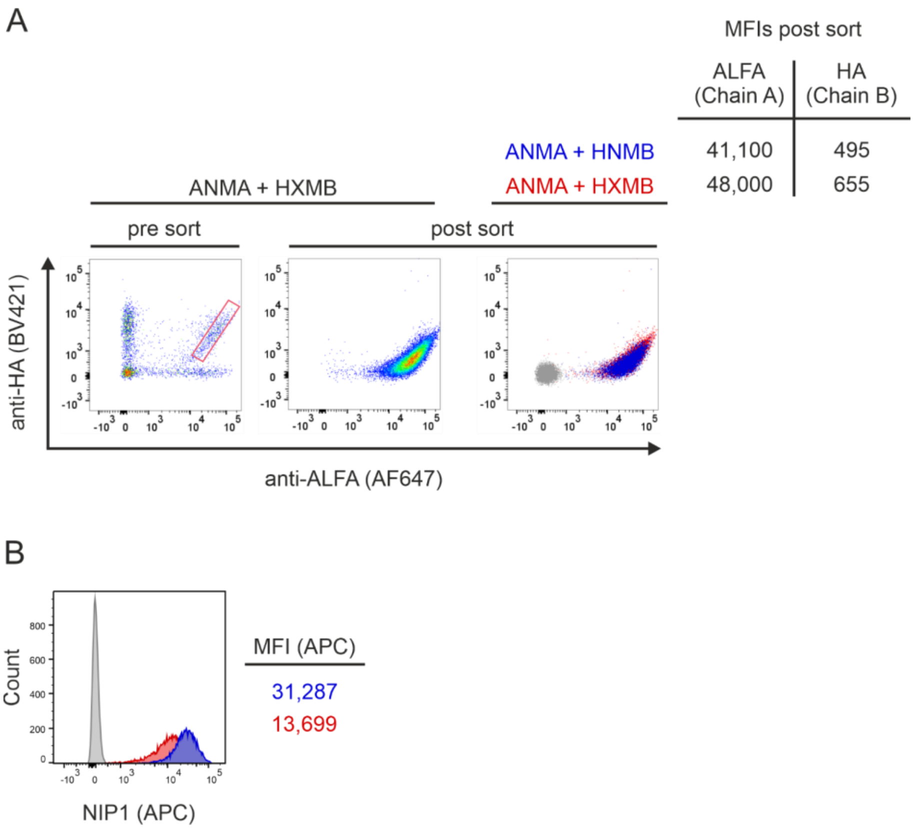

Figures S7. Surface expression of divalent and monovalent mIgM-BCRs. (**A**) Density plots of RHLKO/LC cells that were retrovirally transduced to express the indicated µm HCs. Left plot shows cells after puromycin selection, middle plot shows sorted cells. The overlay plot on the right shows surface expression of divalent (blue) and monovalent (red) mIgM. Mean fluorescence intensities (MFIs) of A and B chains after sorting are given on the right. (**B**) Divalent (blue) and monovalent (red) mIgM-BCRs were stained with a biotinylated NIP1-peptide and APC-conjugated streptavidin.

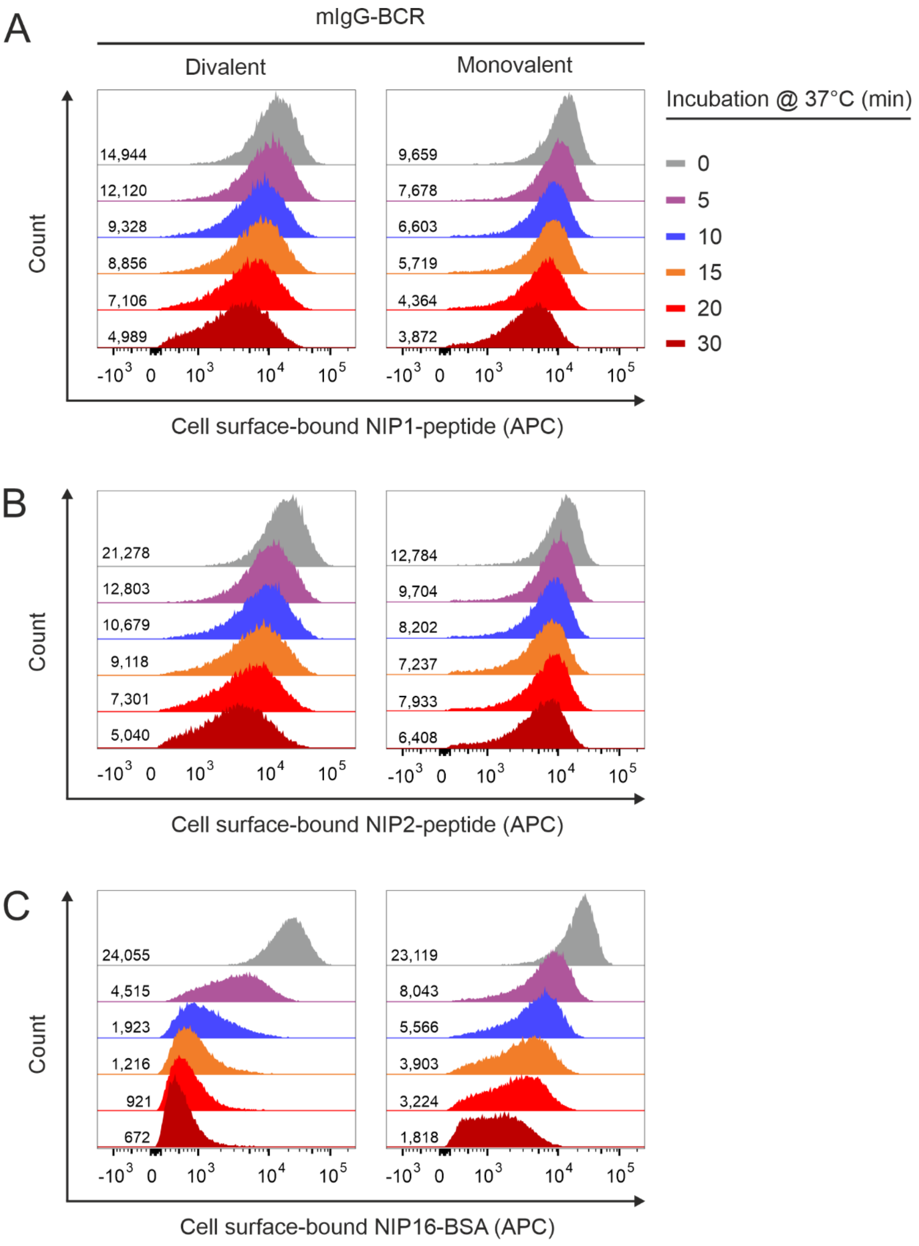

Figure S8. Impaired internalization of monovalent mIgG-BCRs. RHLKO cells expressing either divalent or monovalent NIP-reactive mIgG-BCRs were incubated with a monovalent NIP1-peptide (**A**), a divalent NIP2-peptide (**B**) or polyvalent NIP16-BSA (**C**) for the indicated times at 37°C. Cells that were kept on ice (= time point 0) served as controls. Non-internalized, cell surface-bound antigens were stained with streptavidin-APC and analyzed by flow cytometry. Values in the histograms represent MFIs of APC fluorescence. Statistical analysis of three independent experiments is shown in Figure 4.

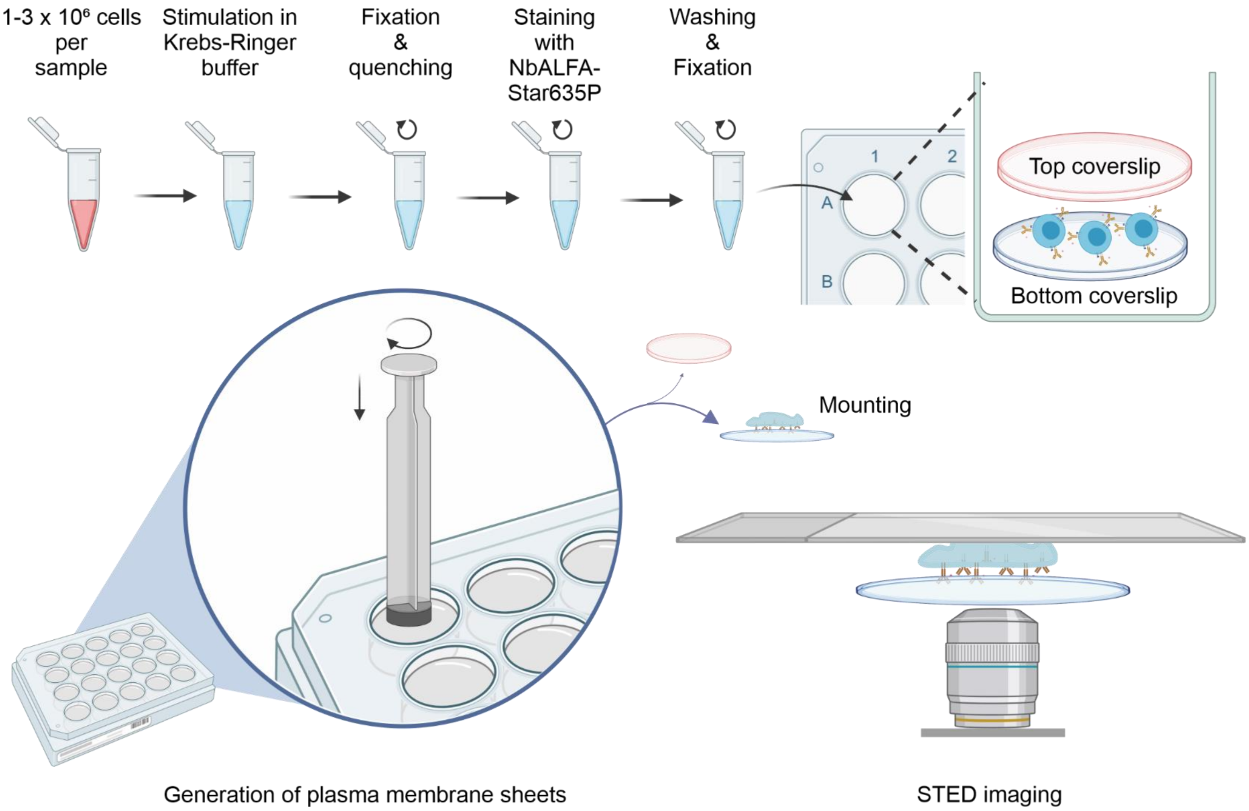

Figure S9. Experimental workflow to generate B cell plasma membrane sheets. Cells were stimulated with antigen in Krebs-Ringer solution containing 1 mM $CaCl_2$, followed by fixation, staining, washing and a second fixation step. Subsequently, cells were transferred onto poly-L-lysine (PLL)-coated coverslips (ø: 18 mm), placed in 12-well plates and centrifuged at 300 × g for 5 minutes to allow for sedimentation of the cells onto the coverslips. A second, clean coverslip was gently placed on top of the cells, creating a coverslip-cell-coverslip 'sandwich'. A flat plunger was then used to press down the coverslip sandwich while rotating the top coverslip clockwise, thus disrupting the cells and flattening the membranes onto the coverslips. Finally, the coverslips were mounted on glass slides for STED imaging.

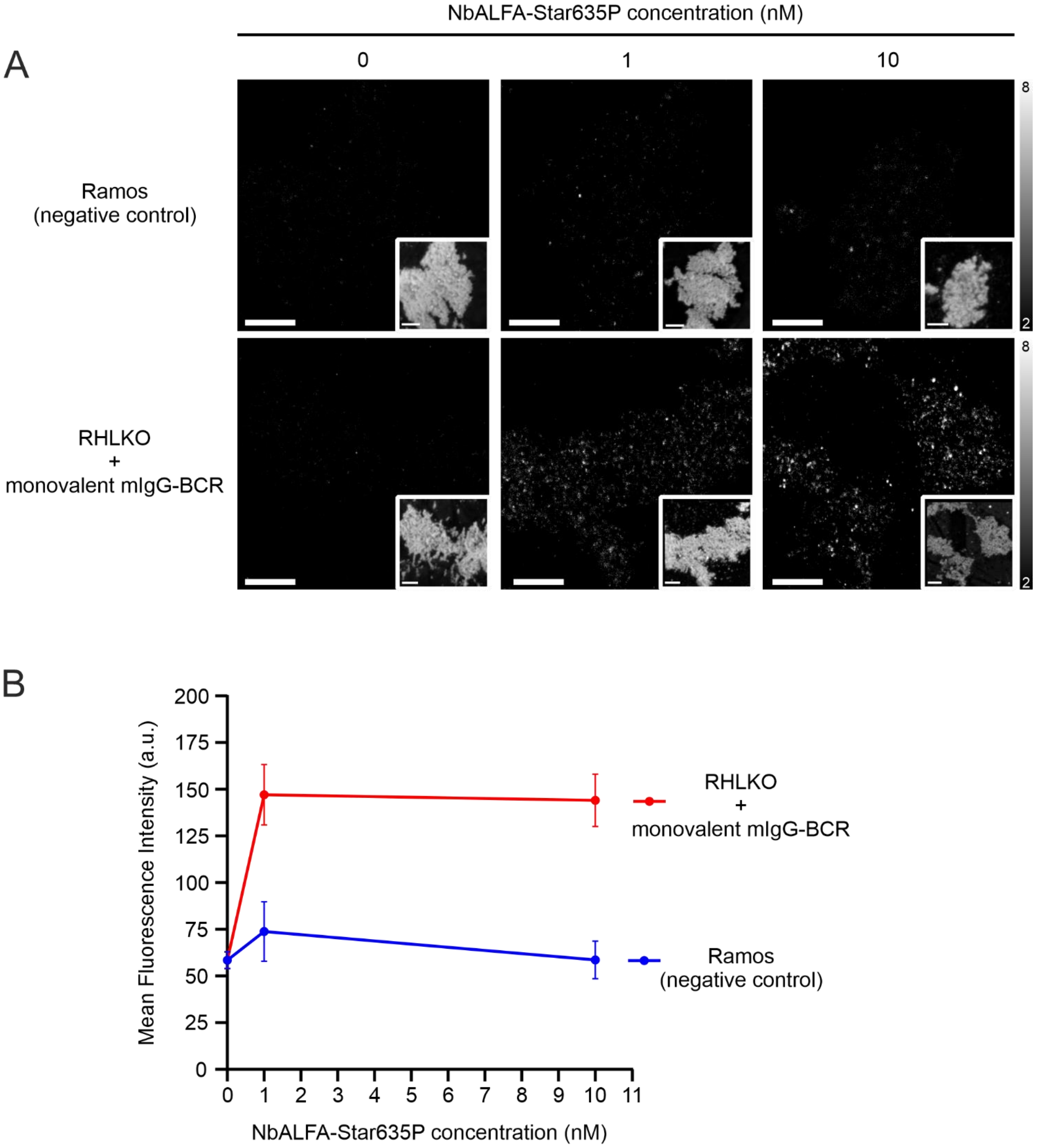

Figure S10. Titration of anti-ALFA sdAb for STED imaging. (**A**) Representative images of plasma membrane sheets. Wild-type Ramos cells (top row, negative control) and RHLKO cells expressing a monovalent mIgG-BCR (bottom row) either were left untreated (left column) or were incubated with either 1 nM (middle column) or 10 nM (right column) concentrations of AbberiorStar635P-conjugated NbALFA. (**B**) Quantifications of the mean fluorescence intensity (MFI ± standard error of the mean (SEM)) of membrane sheets stained with different NbALFA-Q-AberriorStar635P concentrations. Mean fluorescence intensities were obtained using the Image J software. Numbers of analyzed membrane sheets were: Ramos, unstained: 13; 1 nM NbALFA: 6; 10 nM NbALFA: 7; RHLKO + ANGA/HXGB, unstained: 13; 1 nM NbALFA: 10; 10 nM NbALFA: 24. All subsequent staining experiments were carried out with 10 nM of NbALFA-Q-AbberiorStar635P.

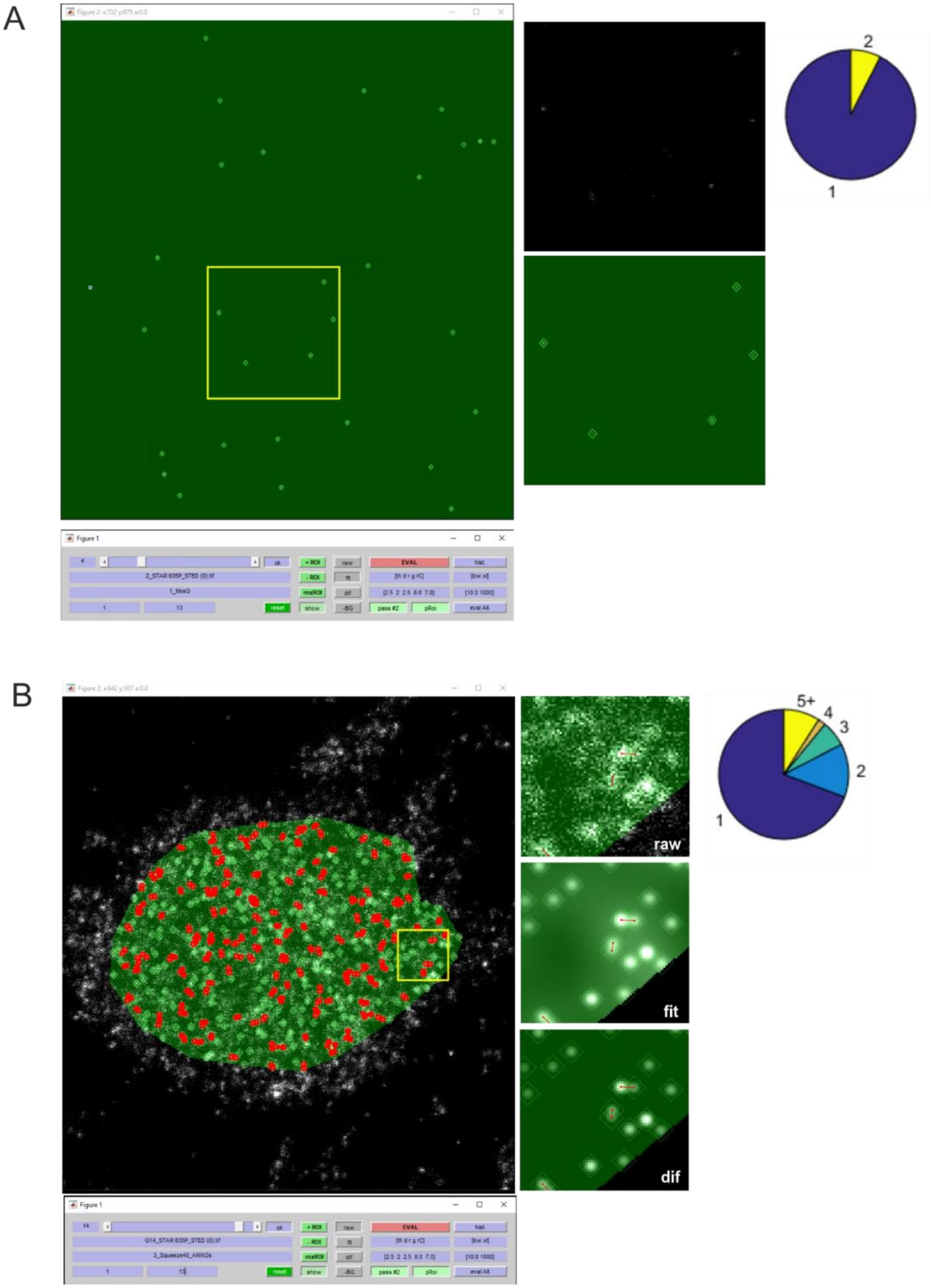

Figure S11. Processing of STED images for fluorescence intensity quantification of spots. (**A**) Representative image of AbberiorStar635P-conjugated anti-ALFA nanobody (NbALFA) dispersed on glass coverslips. The macro defines a reference intensity value corresponding to a single NbALFA-Q-AbberiorStar635P molecule. Pie chart shows the quantification of fluorescence

intensities. The majority of spots contains a single nanobody. (**B**) Representative image of a plasma membrane sheet from cells expressing an ALFA-tagged mIgG-BCR. Membrane sheets were analyzed by selecting a region of interest (ROI), preferably the central area of the membrane sheet, while avoiding the edges, which often contain turned-up regions of membranes. The raw images were processed to identify spots (fit), followed by subtraction of background fluorescence (dif). The data are processed based on the intensity of the detected spots, using the reference intensity unit that was individually determined in each imaging session. Finally, spots were classified as containing groups of 1 to 5+ NbALFA-Q-AbberiorStar635P molecules (equivalent to mIgG-BCRs, which always contain a single ALFA-tag). Data from pie charts were extracted for plotting and analysis.

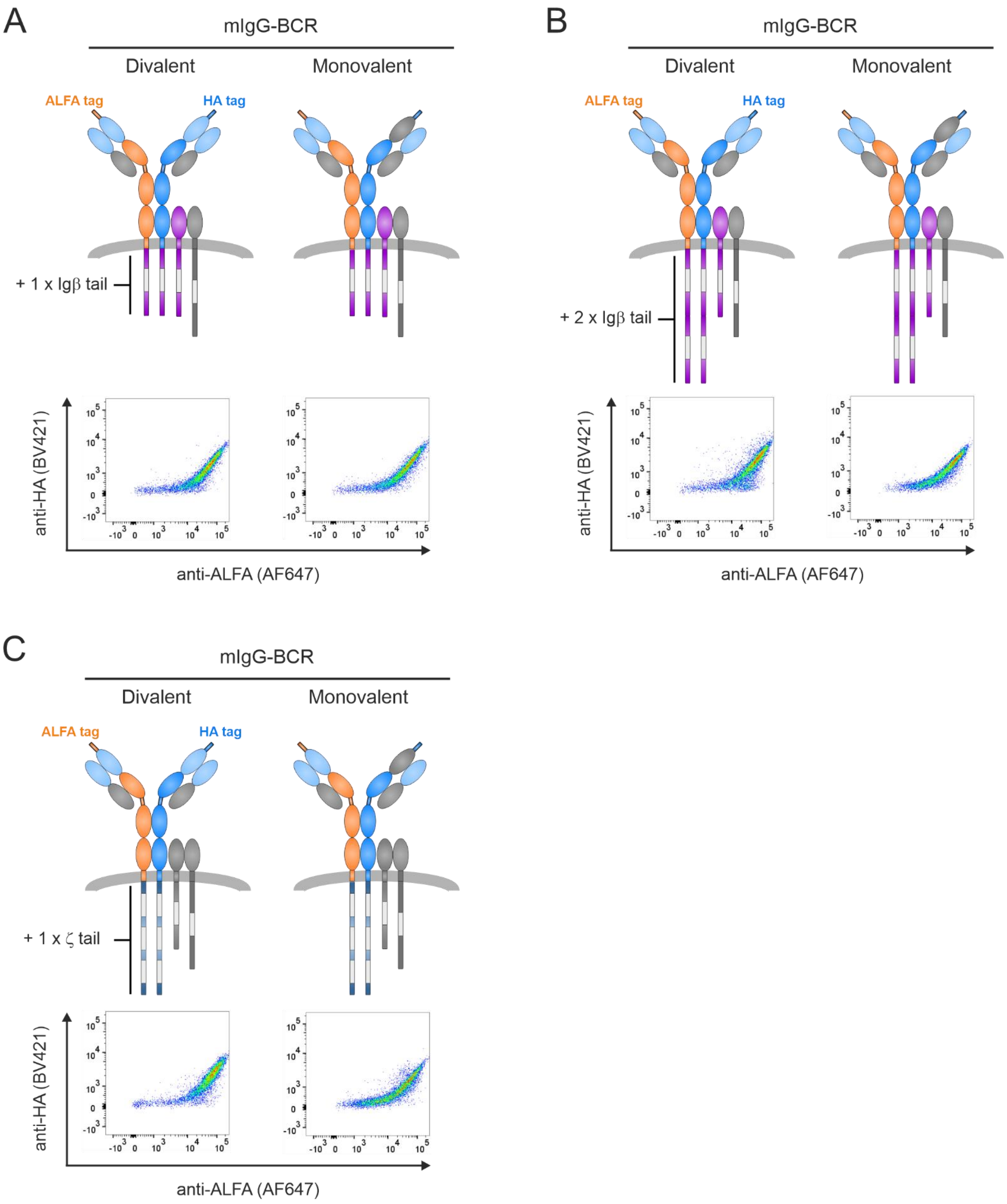

Figure S12. Expression of chimeric mIgG-BCRs containing additional ITAMs. The cytoplasmic domain of Igβ was fused to the transmembrane domain of γ1m chains either as single copy (**A**) or as double copy (**B**). Alternatively, the cytoplasmic domain of the TCR ζ chain was used (**C**). FACS plots show cell surface expression of γ1m A and B chains, stained with anti-ALFA-AF647 and anti-HA-BV421, respectively.

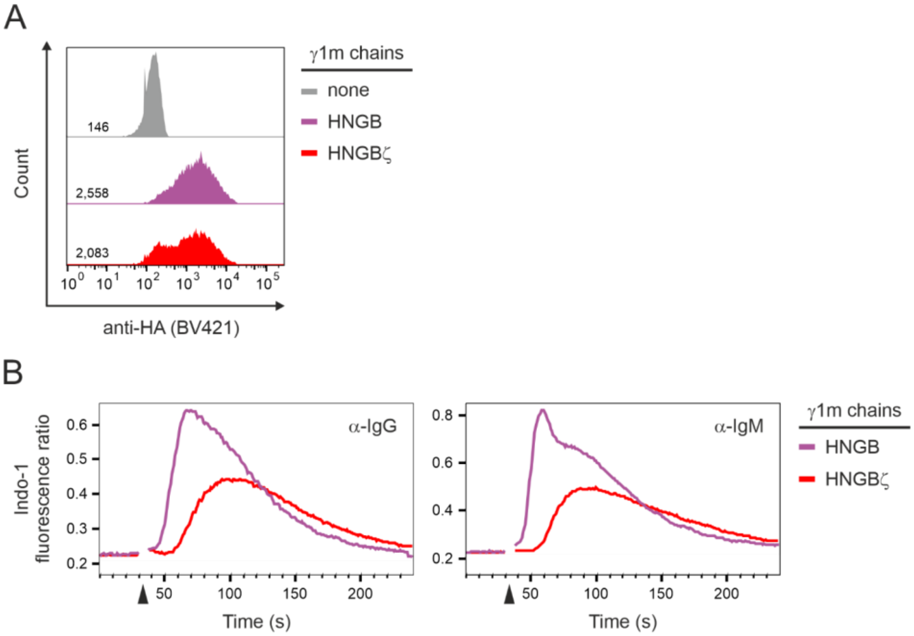

Figure S13. Expression and analysis of mIgG-BCRs with additional ζ chain ITAMs in WEHI-231 B cells. (**A**) Murine WEHI-231 B cells were transfected to express the HNGB chain (purple) or the HNGB chain fused to the TCR ζ chain (red, see Fig. S12C). Cells were sorted twice to obtain homogeneous cell surface expression. (**B**) Ca^{2+} mobilization of cells shown in (A) stimulated either with $F(ab')_2$ against human IgG (α-IgG, left graph) or against mouse IgM (α-IgM, right graph) to activate either exogenous or endogenous BCRs, respectively. Results are representative of three independent experiments.

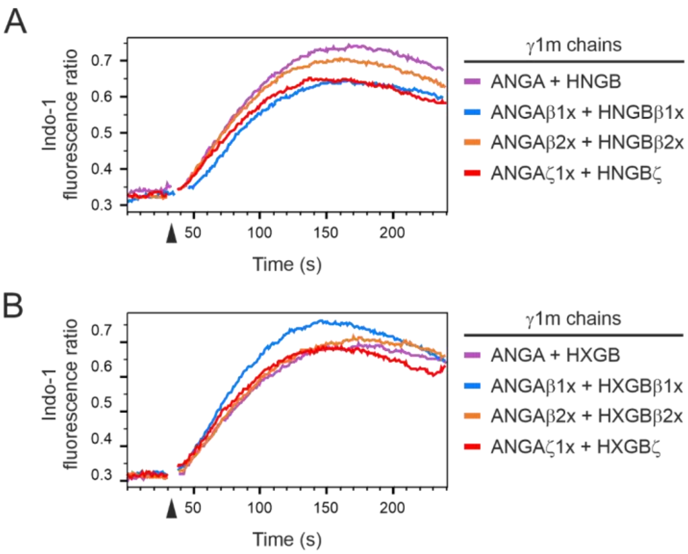

Figure S14. Ca^{2+} store analysis of cells expressing mIgG-BCRs with additional ITAMs. Ca^{2+} mobilization of cells shown in Figure S12 was analyzed on incubation with Thapsigargin. (**A**) Cells expressing divalent BCRs, (**B**) cells expressing monovalent BCRs. Measurements were carried out in Ca^{2+}-free buffer to specifically analyze intracellular Ca^{2+} stores.

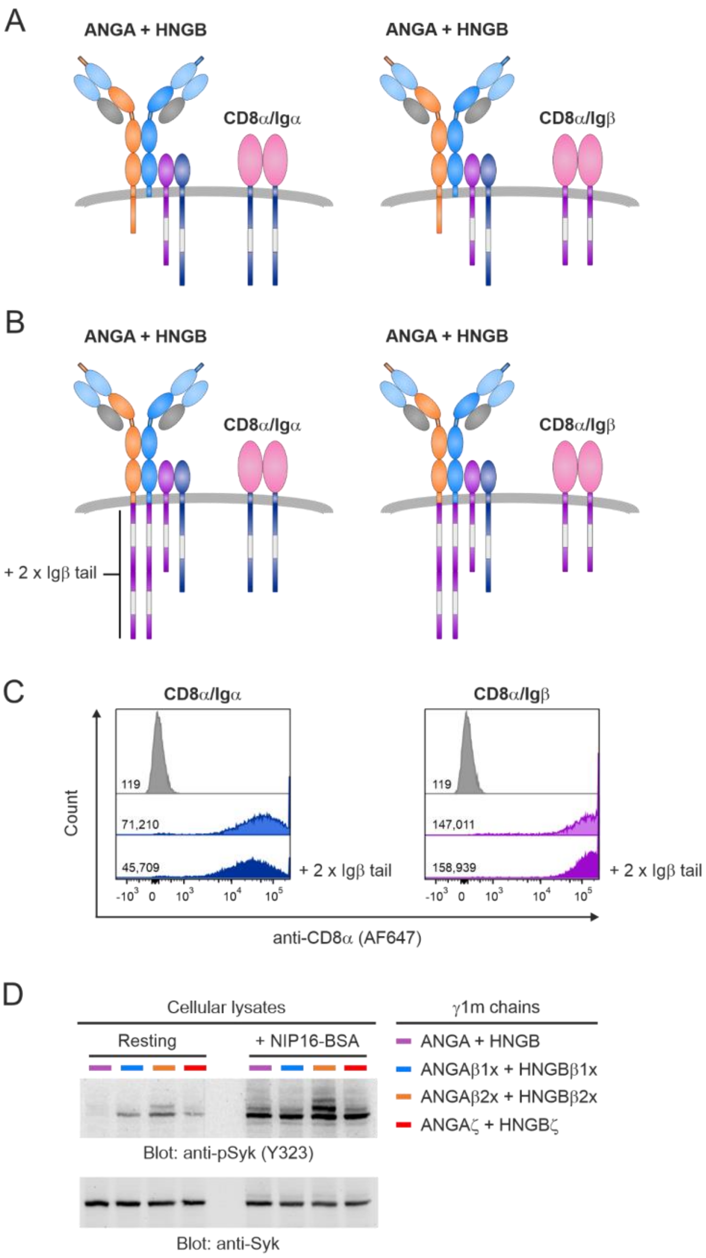

Figure S15. Generation and analysis of cells expressing BCRs with additional ITAMs and chimeric CD8 receptors. (**A**) Schematic depiction of cells expressing divalent mIgG-BCRs and CD8 chimeras containing the cytoplasmic domain of either Igα (blue) or Igβ (purple). (**B**) Same schematic depiction as in (A) showing cells expressing divalent mIgG-BCRs with four additional ITAMs. (**C**) Cell surface expression of CD8 chimeras was analyzed by flow cytometry. Cells were stained with AF647-conjugated anti-CD8 antibodies. (**D**) Tyrosine phosphorylation of Y323 of Syk in the indicated cells was analyzed by immunoblot analysis of lysates from resting cells and cells that had been stimulated with polyvalent NIP16-BSA for three minutes at 37°C. Blot was re-probed with an anti-Syk antibody (clone 4D10) as loading control.

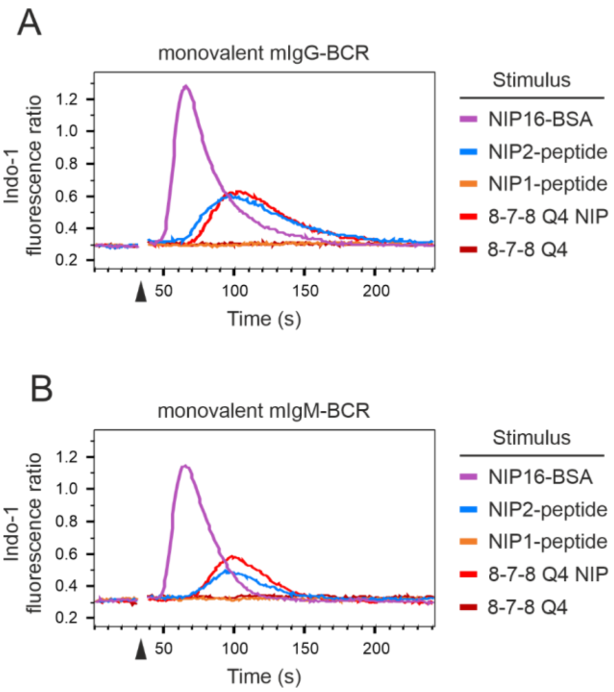

Figure S16. Analysis of Ca^{2+} mobilization induced by monovalent BCRs stimulated with different NIP antigens. Ramos B cells expressing either monovalent mIgG-BCRs (**A**) or monovalent mIgM-BCRs (**B**) were stimulated with the indicated antigens. 8-7-8 Q4 (a carrier without NIP) and 8-7-8 Q4 NIP were kindly provided by Dr. Jørgen Kjems (Aarhus University, Denmark, see Ferapontov et al., 2023, Nat Comms).
